# Tuning the Formation and Growth of Platinum Nanoparticles Using Surfactant: *In Situ* SAXS Study of the Aggregative Growth Mechanism

**DOI:** 10.1021/acsami.5c05268

**Published:** 2025-07-02

**Authors:** Rodolfo Fini, Marina Magnani, Celso V. Santilli, Sandra H. Pulcinelli

**Affiliations:** Institute of Chemistry, 28108 São Paulo State University (UNESP), Araraquara, SP 14800-060, Brazil

**Keywords:** platinum nanoparticles, aggregative growth, surfactant influence, cationic surfactant, hierarchical assembly, electrosteric stabilization, colloidal stability

## Abstract

The synthesis of platinum nanoparticles (Pt NP) *via* chemical reduction with ascorbic acid (AA) and kinetic stabilization with the cationic surfactant tetradecyltrimethylammonium bromide (TTAB) was investigated, with emphasis on the influence of the TTAB/Pt^2+^ ratio on particle size and growth behavior. Based on small-angle X-ray scattering (SAXS), ultraviolet–visible (UV–vis), and transmission electron microscopy (TEM) analyses, a four-stage mechanism was proposed for Pt NP formation, starting from nucleation and initial growth of primary nanoparticles (NP_p_), followed by a hierarchical aggregation process governed by the interplay between attractive and repulsive forces. While the ascorbic acid governs the reduction pathway and remains central to defining the morphology of Pt NP, the addition of TTAB was found to significantly modulate aggregation kinetics and structural organization, even though it does not act as a direct shape-directing agent. The higher the TTAB concentrations, the smaller and more monodisperse the primary NP, the enhanced the electrosteric stabilization, and the denser the aggregates with lower porosity. These changes were closely correlated with a decrease in the aggregation rate and an increase in the activation barrier for aggregation. This work advances the understanding of how cationic surfactants, even when not acting as shape-directing agents, can critically influence the assembly and final architecture of Pt nanostructures, providing valuable insights into the rational design of nanoparticle-based materials.

## Graphical abstract

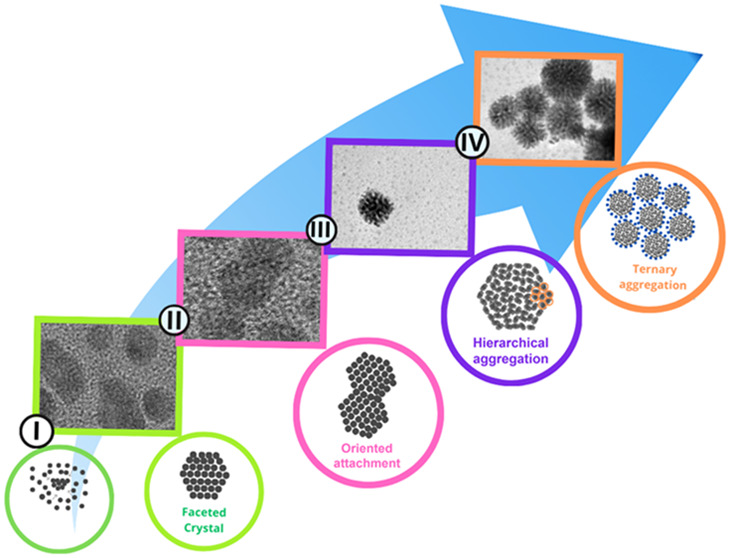

## Introduction

Noble metal nanoparticles (NP) have attracted increasing attention, due to their chemical stability, plasmonic properties, and catalytic applications.^1–3^ Significant efforts have been devoted to tailoring the characteristics of platinum (Pt) NP, with the goal of enhancing their performance and catalytic efficiency, by means of the rational design of diverse nanostructures.^4–10^

Solution-phase synthesis is effective for the controlled production of Pt NP of different shapes and sizes. Several solution-phase synthesis methods use organic molecules as surfactants and structure-directing agents, in combination with chemical reducing agents.^11–14^ For example, it was demonstrated that cubic NP could be obtained from Pt salts, in the presence of tetradecyltrimethylammonium bromide (TTAB) as a surfactant, when sodium borohydride (NaBH_4_) was used as a reducing agent,^15^ while branched NP of various sizes were obtained when the reducing agent was ascorbic acid.^7,14^ Several studies have elucidated the roles of different reducing and shape-controlling agents in the synthesis of metallic nanostructures.^16–18^ However, the actual role of stabilizing agents (such as polymers, surfactants, and other additives) during solution-phase synthesis remains poorly understood.^19^ Most reports have focused on the role of the surfactant in the stabilization of the formed NP (for example, by means of steric or electrostatic effects), ignoring its role in the kinetics of coalescence and growth of NP.^19–21^

Surfactants have crucial effects in the synthesis of metallic NP, with their specific characteristics determining their functions and effects.^22^ In the synthesis of metal oxide nanomaterials, anionic surfactants with negative charges act as templates that stabilize the shape nanoparticles. They play a role in reducing metal precursors and enabling structured growth, influenced by pH.^23–25^ Nonionic surfactants can prevent aggregation and influence nanoparticle shape by selective adsorption.^26,27^ Cationic surfactants derived from organic amines can control morphology by adsorbing on crystal facets, with their ionization in solution affecting the morphological evolution of nanoparticles.^28–30^

Stabilizing agents are frequently employed from the early stages of the reactions since they can control the kinetics of NP growth. They have been found to promote different coordination with Pt complexes and partially stabilize the nanocrystals.^19,31^ While the role of stabilizing agents in nanoparticle synthesis has been broadly explored, their specific impact on the nucleation and growth dynamics of Pt NP remains the subject of ongoing investigation. Angermund et al.^32,33^ suggested that the addition of trialkylaluminum (AlMe_3_) to Pt­(acetylacetonate)_2_, in the presence of citric acid in toluene, could lead to the formation of Pt binuclear complexes, without direct Pt–Pt bonds. O-bridged Pt atoms have been suggested as the embryonic stage for further Pt NP nucleation, although AlMe_3_ can also stabilize the formed NP. The addition of poly­(vinylpyrrolidone) (PVP) and polyamidoamine (PAMAM) can also be effective in stabilizing Pt species in the early stages of reaction.^34,35^ The addition of poly­(vinyl alcohol) (PVA) during the reduction of Pt­(IV) ions with l-ascorbic acid was shown to have a strong effect on the kinetics of the overall process for the formation of Pt NP.^31^ The values of the rate constants for nucleation and growth decreased 2-fold, compared to the system without PVA.

However, further studies are still needed to better understand the role of stabilizing agents, such as cationic surfactants, in the synthesis of Pt NP.^19,31^ While surfactants and other additives are commonly present from the beginning of nanoparticle syntheses—where they influence particle–medium interactions and ultimately affect properties such as size and distribution, shape, and stability^36–40^ —this work aims to further elucidate their role by specifically investigating how a cationic surfactant, commonly used in Pt NP synthesis,^15,41^ modulates the rate and efficiency of aggregation. These factors can have a significant impact on final particle characteristics, including porosity, which in turn may influence material performance in applications, such as catalysis.

A previous study by Seo et al.^41^ demonstrated that the alkyl chain length and concentration of cationic surfactants such as cetrimonium bromide (CTAB), and tetradecyltrimethylammonium bromide (TTAB), can significantly influence the size and the shape of Pt nanoparticles reduced with NaBH_4_. These effects were mainly attributed to interactions between Pt precursor complexes and surfactant micelles, which modulate the nanoparticle growth process. For TTAB, the authors observed that increasing the surfactant concentration led to a decrease in particle size, while favoring the formation of cuboctahedral shapes. However, the growth mechanism or reaction kinetics, which could further elucidate the origin of these morphological characteristics, were not investigated in this study. It has also been shown that anisotropic Pt nanostructures, such as single-crystal hyperbranched morphologies, can be synthesized without the use of surfactants or seeds, as the byproducts of ascorbic acid oxidation (notably 2,3-diketo-l-gulonic acid) can act as shape-directing agents.^14^

While these previous studies^14,41^ have investigated how ascorbic acid or surfactants like TTAB influence particle morphology, few have explored their combined effects on the aggregation kinetics and structural hierarchy of Pt nanoparticles. Moreover, most reports^14,41^ rely on *ex situ* techniques, which limit the temporal resolution of mechanistic insights. In this work, we bridge this gap by applying *in situ* SAXS to track, in real time, the formation, aggregation, and structural evolution of Pt NP under varying TTAB concentrations, offering a unique kinetic perspective on nanoparticle assembly.

In this context, the present work investigates the role of the cationic surfactant TTAB, used as a model cationic surfactant, in the mechanism of aggregative growth of platinum nanoparticles. The gyration radius (*R*_g_) and the number of particles (N) across different nanoparticle size populations were determined experimentally using time-resolved *in situ* small-angle X-ray scattering (SAXS), enabling the analysis of how TTAB concentration affects the kinetic pathway. Complementary qualitative evaluation of particle morphology and quantitative evaluation of the size distribution was conducted by *ex situ* transmission electron microscopy (TEM). The results demonstrated that tuning the surfactant concentration allows the modulation of the aggregation kinetics and, consequently, of NP nanostructure evolution.

## Experimental Section

### Materials

The following chemicals were used as received: tetradecyltrimethylammonium bromide (TTAB, 99%, CAS 1119–97–7, Sigma-Aldrich), potassium tetrachloroplatinate­(II) (K_2_PtCl_4_, 99.9%, CAS 10025–99–7, Sigma-Aldrich), and ascorbic acid (AA, 98%, CAS 50–81–7, Sigma-Aldrich). The water used had a minimum resistivity of 18 MΩ·cm at 25 °C.

### Pt NP Synthesis Procedure

Synthesis of the Pt NP was based on the procedure reported elsewhere^14,15^ with adaptations. For this, 5 mL of a K_2_PtCl_4_ solution (10 mmol·L^–1^) was mixed with variable volume (*x* = 18.75, 12.5, 6.25, or 0 mL) of a TTAB solution (400 mmol·L^–1^) in a round-bottom flask. The total volume was adjusted to 47 mL of deionized water. In all systems, the concentration of Pt^2+^ was maintained at 1 mmol·L^–1^, while the TTAB concentration was varied to 150, 100, 50, and 0 mmol·L^–1^, corresponding to the samples denoted 150AA, 100AA, 50AA, and 0AA, respectively. The mixture was stirred for 10 min at room temperature and for an additional 10 min at 70 °C. Subsequently, 3 mL of ascorbic acid solution (500 mmol·L^–1^) was added to the closed system. The reaction mixture was stirred for 12 h at 70 °C, leading to the formation of a dark colloidal suspension.

### *In Situ* Characterizations

*In situ* small-angle X-ray scattering (SAXS) experiments were performed to follow the formation and growth of the Pt NP synthesized by reduction with AA, using the SAXS1 beamline of the Brazilian National Synchrotron Light Laboratory (LNLS, Campinas, São Paulo). An asymmetrically cut and bent Si(111) crystal was used to horizontally focus the monochromatic X-ray beam (λ = 0.1544 nm). The scattering intensity, *I*(*q*), measured with a two-dimensional X-ray detector (PILATUS 300 K, Dectris), was obtained as a function of the modulus of the scattering wave vector, *q* = (4π/λ)­sin­(θ/2), where θ is the scattering angle, and λ is the wavelength. The distance between the sample and the detector was fixed at 887.83 mm, corresponding to a q range between 0.13 and 5 nm^–1^. Measurements began before the electronically controlled injection of the AA solution into the reaction mixture and were continued for 2 h thereafter. SAXS curves were collected every 30 s, 29 s of acquisition time, and 1 s in which the beam was shut, during all of the synthesis steps. To perform the SAXS measurements, the reaction solution was kept at 70 °C and sent to the sample holder in a closed loop, at a constant flow rate of 10 mL·s^–1^, using a peristaltic pump. A schematic representation of the *in situ* measurements is provided in the Supporting Information (Figure S1), which illustrates the experimental setup for *in situ* data acquisition using the ultraviolet–visible (UV–vis) and SAXS techniques. An inline UV–vis probe was used to collect data directly from the reaction medium. To ensure continuous data collection throughout the entire nanoparticle synthesis, ascorbic acid was added to the reaction medium only after both SAXS and UV–vis coupled data collection had started.

Sample 150AA was also chosen to be analyzed by coupled UV–vis spectroscopy once it showed the slowest growth kinetics, allowing to clearly distinguish the UV–vis evolution data. To UV–vis spectroscopy measurements, a Cary 60 instrument (Agilent) coupled to a UV–vis probe (10 mm path length) was used in the reaction medium. The UV–vis spectra were acquired every 30 s at a resolution of 1 nm in the range 200–500 nm. The background signal was obtained by using water as a blank.

### SAXS Data Evaluation

The SAXS curves were used to obtain the gyration radius (*R*_g_) and the scattering intensity, as *q* → 0 (*I*_0_), using the Guinier law^42^ (eq 1). *I*_0_ depends on the number density of scattering objects (*N*), the squared electronic density difference between scattering phases (Δρ^2^), and the squared volume of the scatterers, making it directly proportional to *R*_g_^6^, as indicated by eq 2. To fit the SAXS curves using eq 1, *I*(*q*) was subtracted by *I*(*q*) at *t* = 0 min to disregard the contribution of the TTAB micelles for samples containing TTAB. The synthesis performed in the presence of TTAB showed a maximum at high q values with two Guinier regions at around 0.3 and 0.1 nm^–1^. The *R*_g,*x*_ was calculated for two distinct families of scatterers: family 1 (NP_1_) and family 2 (NP_2_), appearing at around *q* ∼ 0.1 nm^–1^ and *q* ∼ 0.3 nm^–1^, resulting in *R*_g,1_, *R*_g,2_, *I*_0,1_, and *I*_0,2_, respectively.

1 $$I\left(q\right)=I_{0}\cdot \exp\left(-\frac{R_{g}^{2}}{3}\cdot q^{2}\right)$$

2 $$I_{0}\propto N\cdot \Delta \rho^{2}\cdot R_{g}^{6}$$

Assuming that the overall electronic density difference remained constant at each level, *I*_0_ could be normalized with *R*_g_^6^, yielding eq 3, giving a value proportional to the number of scatterers, denoted as *N*_*x*_. From this equation, it was possible to obtain a value proportional to the number of particles from families 1 and 2 (*N*_1_ and *N*_2_, respectively).

3 $$\frac{I_{0}}{R_{g}^{6}}\propto N_{x}$$

The maximum located at 1 nm^–1^ < *q* (nm^–1^) < 3 nm^–1^ for the samples synthesized with TTAB was assumed to be associated with the presence of spatially correlated primary platinum nanoparticle (NP_p_), formed by aggregates of hyperbranched Pt NP. In other words, the assumption was made of a three-level hierarchical structure model, with the first (finer) level consisting of correlated primary Pt NP, while the second and third levels corresponded to coarse aggregates embedded in the liquid medium. The SAXS intensity produced by an isotropic system of isolated nanoparticles embedded in a homogeneous matrix, with spatial correlation and uncorrelated orientation, can be described by a semiempirical function proposed by Beaucage^43^ (eq 4), where *R*_g,p_ is the Guinier gyration radius of the NP_p_, while *G*_0,p_, *B*, and *P* are adjustable parameters, and the ratio *G*_0,p_/*B* depends on the nanoparticle structure and shape.

4 $$I\left(q\right)=\left[G_{0,p}\cdot \exp\left(-\frac{R_{g,p}^{2}}{3}\cdot q^{2}\right)+B\cdot {\left(\frac{{\left(\mathrm{erf}\cdot \left(\frac{q\cdot R_{g,p}}{\sqrt{6}}\right)\right)}^{3}}{q}\right)}^{P}\right]\cdot S\left(q\right)$$

Beaucage^43^ also derived a simple equation, using the Born–Green approximation, for the isotropic structure–function, *S*(*q*), given by eq 5, where k is a parameter known as the packing factor and Θ­(*q*) is a function given by eq 6, where d is the average distance between nanoparticles. Equation 6 was derived for systems of identical spatially correlated nanoparticles, but it is usually also applied to systems of correlated particles with a narrow or moderate width size distribution.^44^

5 $$S\left(q\right)=\frac{1}{1+\kappa \cdot \Theta \left(q\right)}$$

6 $$\Theta \left(q\right)=\frac{3\cdot \left[\sin\left(q\cdot d\right)-q\cdot d\cdot \cos\left(q\cdot d\right)\right]}{q\cdot d^{3}}$$

To fit the SAXS curves for the NP_p_, *I*(*q*) was also subtracted by *I*(*q*) at *t* = 0 min, once the main contribution for the SAXS curve is in the region where primary NP are observed. During data evaluation for the NP_p_ level, the Porod exponent was fixed at 4 to reflect smooth interfaces, while the remaining parameters were allowed to vary within a physically meaningful range. Further details are presented in SI.

Similar to *I*_0_, the Guinier prefactor, *G*_0,p_, depends on the values of *N*, Δρ, and volume of the scattering particles. Therefore, a value proportional to the number concentration of primary particles (*N*_p_) can be approximated by using eq 7.

7 $$\frac{G_{0,p}}{R_{g,p}^{6}}\propto N_{p}$$

The ratio between the *N*_p_ and the number concentration of aggregates (*N*_*x*_), measured after a period of aggregation, allows estimation of the average aggregate size—expressed as the average number of primary particles per aggregate—and thus serves as an indicator of the mean aggregate volume.^45^ To further investigate the structural characteristics of the nanoparticles and the influence of TTAB within the aggregates, a parameter proportional to the density of primary particles within each aggregate (d*N*_p_) was calculated. This was done by normalizing the *N*_p_/*N*_*x*_ ratio by the cube of the radius of gyration (*R*_g,*x*_^3^), as shown in eq 8. This approach provides valuable insight into the degree of compaction or porosity of the nanoparticle aggregates.

8 $$\frac{N_{p}}{N_{x}\cdot R_{g,x}^{3}}\propto dN_{p}$$

### *Ex Situ* Characterization

Transmission electron microscopy (TEM) measurements were performed using a JEOL JEM-2100F instrument, available at the Brazilian National Nanotechnology Laboratory (LNNano-CNPEM), to qualitatively evaluate the morphology and quantitatively evaluate the size distribution of the Pt NP. For TEM analysis, a drop of diluted colloidal suspension was deposited on a copper grid, and the water was allowed to evaporate. The samples were evaluated 12 h after the end of 120 min of *in situ* monitoring.

UV–vis analyses were performed using an Agilent Cary 60 instrument in the range 200–800 nm, with 1 nm resolution, at a scanning speed of 1 nm·s^–1^. The samples studied *ex situ* were TTAB (400 mmol·L^–1^), K_2_PtCl_4_ (10 mmol·L^–1^), and TTAB + K_2_PtCl_4_ (TTAB/Pt^2+^ ratio = 100).

X-ray absorption spectroscopy (XAS) measurements were performed at the L_3_ edge of platinum (11,564 eV), at the XAFS-2 beamline of LNLS, using fluorescence mode. The normalization parameters were threshold energy (*E*_0_) of 11,564 eV, pre-edge range 11,462–11,504 eV, postedge range 11,714–12,264 eV, and normalization function of order of 3. The Pt foil reference and the 100AA sample were measured at least three times, for better statistics, and Athena software^46^ was used to calibrate the reference. The extended X-ray absorption fine structure (EXAFS) oscillations, χ­(*k*), were extracted from the data, as a function of photoelectron wavenumber, *k*, and were Fourier transformed using a Kaiser–Bassel function for the real and reciprocal space with *dk* = 2. The theoretical paths were generated using FEFF8 and the modeling was performed in the conventional way, using ARTEMIS with FEFF8 built into it.^46^ Fitting parameters for the first shell were obtained by modeling the EXAFS data of each sample in *R*-space until a satisfactory fit describing the system was obtained. Amplitude (*S*_0_^2^) was obtained from fitting for Pt foil and was used to fit the metallic sample. The *S*_0_^2^ for K_2_PtCl_4_ was also fitted and was used to fit the solution of TTAB + K_2_PtCl_4_.

## Results and Discussion

### SAXS Evolution of Different Pt NP Families

Figure 1a–d shows the temporal evolution of the SAXS curves obtained *in situ* during the platinum synthesis, for the 0AA, 50AA, 100AA and 150AA samples, respectively. The SAXS curves shown here were subtracted by water scattering profile, as our aim was to highlight the contribution of the micelles to the SAXS signal—especially evident at the early stages of the synthesis. Additionally, the SAXS curves subtracted by the TTAB solution resulted in noisy curves during this initial period. For comparison, Figure S2 in the SI presents SAXS data, from a representative stage of the synthesis of 150AA, along with further details on the SAXS data fitting procedure. At the beginning of the synthesis, two maxima corresponding to the form factor of micellar structures^47^ occurred at *q* > 0.3 nm^–1^ for the samples containing TTAB (Figure 1). As time progressed, the contribution of micelles to the overall pattern decreased as the size and number of the scattering Pt NP increased. Furthermore, the decay in the intermediate q range (0.3–1.0 nm^–1^ for the samples synthesized with TTAB) was in accordance with the Porod power-law behavior (*I*(*q*) ∝ *q* ^a^). The value slightly smaller than −4 (∼−3.8), obtained for the 50AA, 100AA and 150AA samples, was characteristic of a biphasic system with a rough interface. The presence of *q*_p_, the correlation peak, which indicates the presence of interacting particles in a packed system, is marked by arrows in the SAXS curves. For the 0AA sample, the correlation peak was absent, but a plateau followed by an exponential decay (Guinier region) occurred for *q* > 0.06 nm^–1^, from which *R*_g,p_ and *I*_0_ for the NP_p_ were also calculated using the Guinier law. In the higher q region (*q* > 1.6 nm^–1^), the decay of *I*(*q*) predicted by the Porod power law indicated the formation of dense particles, with *a* = −3.7. In addition, a second power-law decay with *a* = −3.9 occurred in the low q range (*q* < 0.3 nm^–1^), suggesting the presence of dense aggregates, which was confirmed by the TEM images (presented in Figures 5 and in S4).

**Figure 1:**
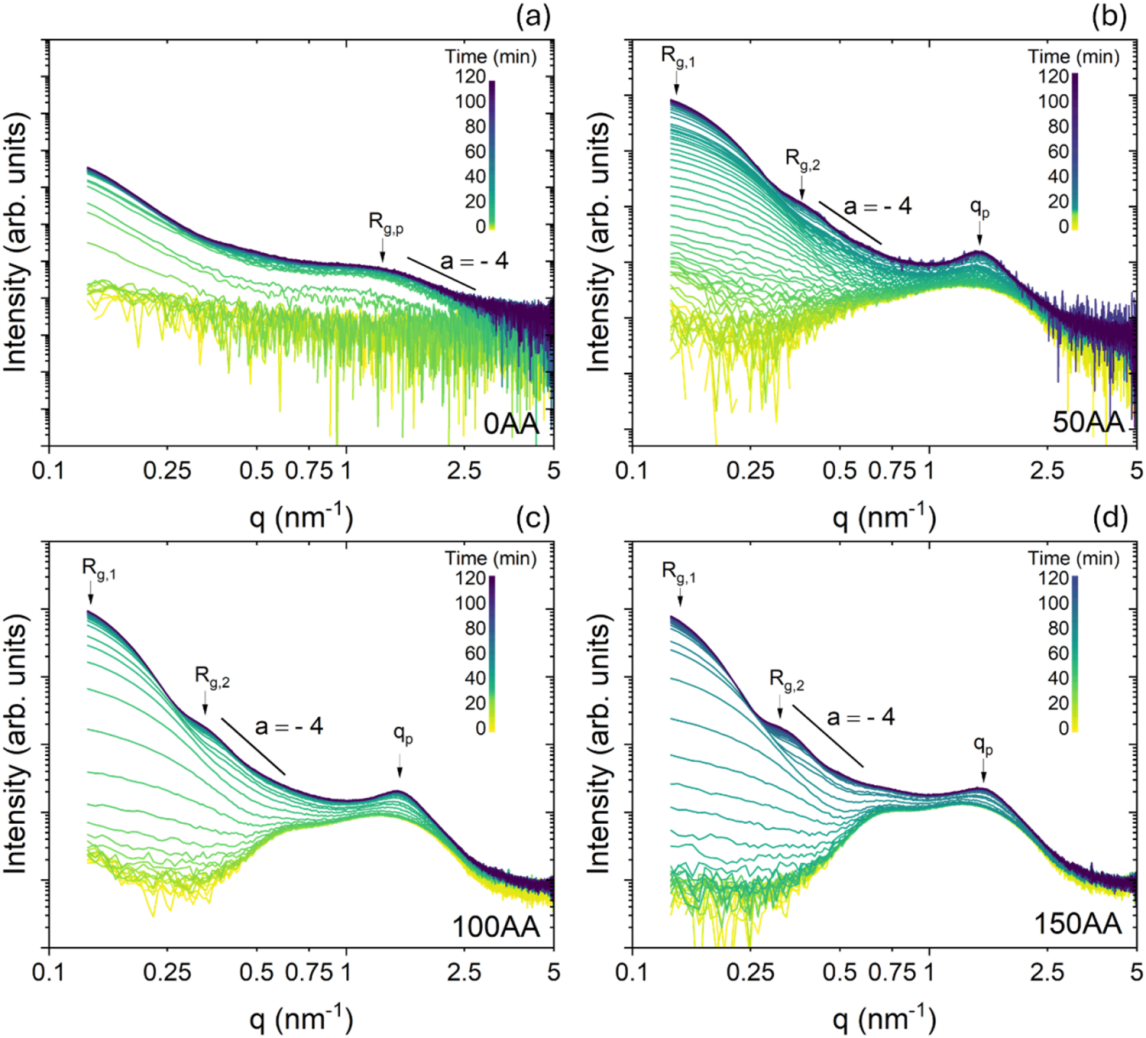
Temporal evolution of *in situ* SAXS curves during the platinum synthesis for samples (a) 0AA, (b) 50AA, (c) 100AA, and (d) 150AA. The lines with a value of *a* = −4 are shown for comparison.

It should be noted that neither the power-law behavior nor the Guinier plateau was observed in the initial stages of the experiment, indicating the occurrence of an incubation period (Δ*t*_i_). This Δ*t*_i_ value was defined as the earliest time point at which a reliable radius of gyration could be determined. The time between the addition of the reduction agent and the appearance of the first particle could be attributed to a nucleation stage and increased progressively with the amount of TTAB, varying from 9 to 52 min for samples 0AA to 150AA, respectively (Figure 2). During this initial period, only the scattering pattern of the micelles was observed, as discussed further below.

**Figure 2:**
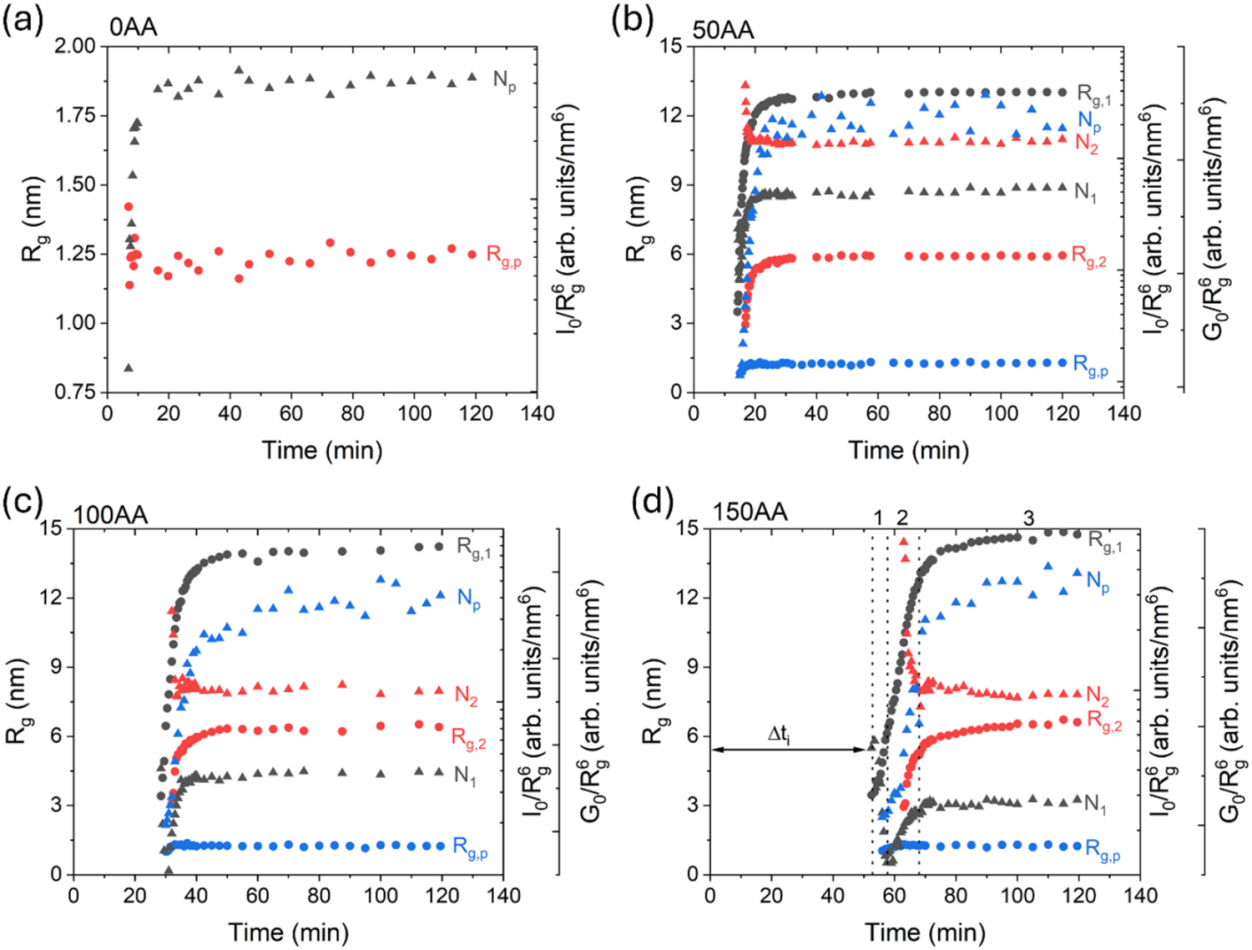
Temporal evolution of *R*_g_ (circles) and N (triangles) for the different families of particles, for samples (a) 0AA, (b) 50AA, (c) 100AA, and (d) 150AA. (d) also indicates the different stages observed during the synthesis of the nanoparticles.

The temporal evolutions of *R*_g,1_, *R*_g,2_, *I*_0,1_, and *I*_0,2_ (Figure 2) were obtained using eq 1, while *R*_g,p_ and *G*_0,p_ were obtained using eq 4. Figure 2a shows the evolution of *R*_g,p_ and *I*_0_/*R*_g,p_^6^ for the 0AA sample in which the number of scatterers (*I*_0_/*R*_g_^6^ ∝ *N*) increased rapidly during the first 20 min, while *R*_g,p_ remained approximately constant, characterizing the nucleation of NP_p_. Due to the absence of Gaussian decay at low angles, it was not possible to apply the Guinier law to calculate, from the SAXS curves, the sizes of larger objects during this step of the synthesis. Figure 2b–d shows the temporal evolution of *R*_g_ and *N* or *N*_p_ (*I*_0_/*R*_g_^6^ or *G*_0_/*R*_g_^6^) for the samples prepared with different amounts of TTAB. All of the samples prepared with TTAB presented different time regimes, which could be more easily observed for the 150AA sample, where the changes occurred in a wider time window. During the first step, after Δ*t*_i_ (∼53 min), a reduction of *N*_1_ and an increase of *R*_g,1_ from 3.4 to 6.6 nm (Figure 2d) were clearly observed for sample 150AA, indicating self-aggregative growth (coalescence) of the smaller nanoparticles formed by the NP_p_. In fact, NP_p_ was not observed by SAXS during the initial period of measurements, mainly due to the TTAB scattering, but the presence of NP_p_ was confirmed by *in situ* UV–vis spectra collected during the early stage of the synthesis (Figure 3). In a second stage (53 < time (min) < 75 for the 150AA sample), *R*_g,1_ and *N*_1_ increased, while a new family of size *R*_g,2_ and number *N*_2_ also occurred, with *N*_2_ decreasing to a minimum at 67 min (marked by the vertical dashed line) and *R*_g,2_ increasing from 2.7 to 6.0 nm. In the advanced stage of the synthesis (75 < time (min) ≤ 120), *N*_1_ and *N*_2_ decreased slowly, while *R*_g,1_ and *R*_g,2_ increased slowly and continuously. In this later step, the growth of *R*_g,1_ and *R*_g,2_ could be explained by a less effective aggregation process. TEM analyses (Figure 5c) support the interpretation that the larger scatterers detected by SAXS are dense aggregates.

**Figure 3:**
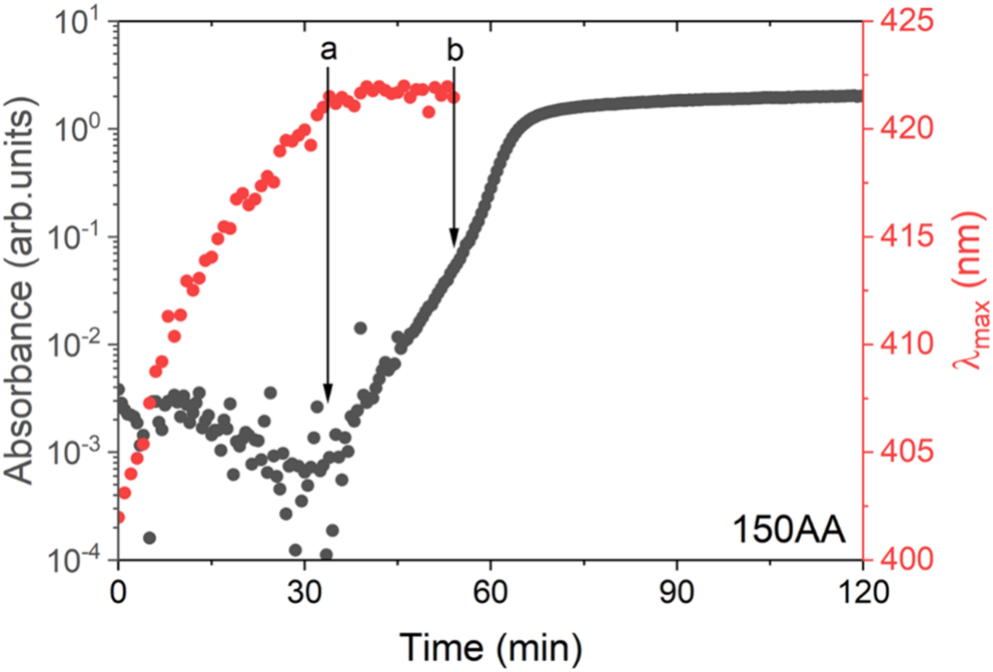
*In situ* evolution of the logarithm of absorbance at 500 nm and the shift in λ_max_.

In summary, all the samples prepared with TTAB presented a similar trend, with three main steps: (1) *N*_1_ decreased, while *R*_g,1_ increased; (2) *N*_1_ increased, while *R*_g,1_ initially increased rapidly and then decelerated. *N*_2_ decreased, reaching a minimum at around 67 min, while *R*_g,2_ increased for sample 150AA, indicating a second aggregate growth. This successive aggregative growth could provide an explanation for the hierarchical structure observed by TEM (Figure 5b); (3) In a complex step, *R*_g,1_ and *R*_g,2_ increased slowly, *N*_1_ and *N*_2_ remained virtually constant, *R*_g,p_ remained constant, and *N*_p_ increased, indicating constant addition of NP_p_ to the existing aggregates.

### Incubation Period and Nucleation of the Condensed Phase

The platinum species present in the 150AA solution during the incubation period were analyzed by UV–vis spectroscopy. The temporal evolution of the UV–vis spectra for the 150AA sample is shown in Figure S3a, while Figure S3b highlights the two maxima, at 326 and 404 nm, observed in the first minutes of the synthesis. The maximum at ∼400 nm, characteristic of the [PtCl_4_]^2+^ complex, shifted to 425 nm, indicating the transformation of this complex to [PtBr_4_]^2+^ during the incubation period.^48^ The baseline increased over time due to the formation of black particles that absorbed at all wavelengths. These features were consistent with the EXAFS data (Figure S3c,d and Table S1) revealing a shift in the Pt^2+^ first coordination shell distance from 2.30 to 2.43 Å, due to the larger size of bromide, compared to chloride ligands. In addition, the decrease of the coordination number for the platinum nanoparticle first coordination shell, relative to platinum foil, was an effect of the smaller nanoparticle size.

Although no information was obtained from the SAXS curves during the incubation time, Δ*t*_i_, the evolution of the UV–vis features (Figure 3) indicated the formation of NP_p_. During the first 35 min of the synthesis, the logarithm of the absorbance at 500 nm remained almost constant, indicating that there was no formation of nanoparticles, since black nanoparticles of platinum should absorb light in the visible spectrum, as reported elsewhere.^49^ On the other hand, the observed red shift of the absorption band evidenced the exchange of chloride by bromide in the Pt^2+^ coordination shell.^48^ After this period, there was an increase in the logarithm of the absorbance in the time window a–b, shown in Figure 3, indicating the formation of NP_p_. The UV–vis spectra baseline increased slowly, until reaching the point indicated by b, where an accelerated increase in the logarithm of the baseline occurred, which coincided with the rapid evolution of *R*_g,1_ (Figure 2d, 55 min < time < 75 min). The trend observed in this data set indicated that during Δ*t*_i_, two phenomena occurred successively. First, there was the formation of the [PtBr_4_]^2+^ complex, until the time corresponding to point a, followed by the formation of the primary NP, which increased until reaching a concentration at which the NP_p_ was no longer stable, giving rise to a burst process of growth by aggregation.

### First Aggregation Step

In order to account for interactions of NP_p_ in a packed system, the *R*_g,p_ obtained by fitting the SAXS data with eq 4 was adjusted with the packing factor, **κ**, defined in eq 5. As shown in Figure 4, after the induction time, Δ*t*_i_, the packing factor increased rapidly from zero to a value close to 6. The increase in the TTAB amount increased Δ*t*_i_, without causing changes in the packing of particles. It should be noted that eq 4 includes a term to account for multiple particle correlations (*S*(*q*)), which is effective for weak correlations of any type but becomes more restricted for spherical correlations.^44^ As the correlations become stronger, its strength (κ = 8v_H_/v_O_) varies from 0 (no correlations) to about 5.9, indicative of FCC (face-centered cubic) or HCP (hexagonal close-packed) structures.^50^ Therefore, it could be concluded that for the *x*AA (*x* = 50, 100 or 150) samples, NP_p_ formed particle aggregates of families 1 and 2 (*R*_g,1_ and *R*_g,2_), where the size of the *R*_g,p_ remained constant, while *R*_g,1_ and *R*_g,2_ increased.

**Figure 4:**
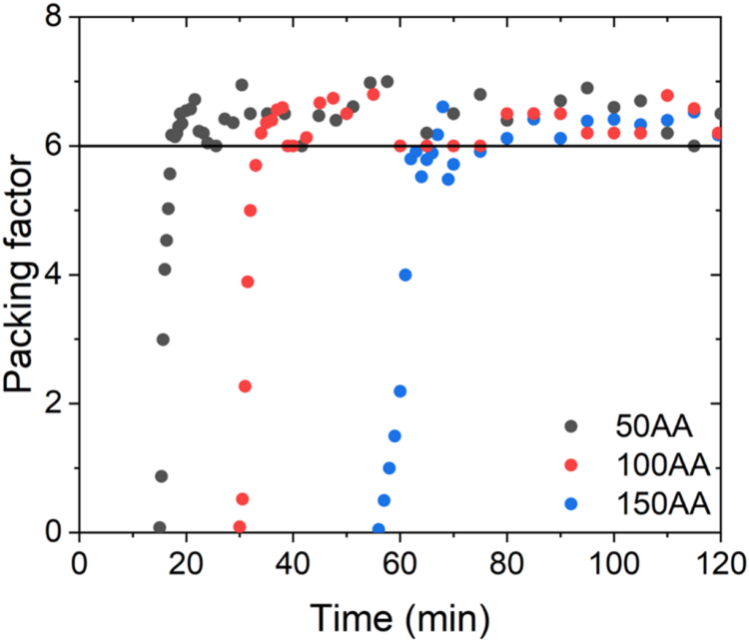
Temporal evolution of the packing factor for samples prepared in the presence of different amounts of TTAB.

The particle packing results were corroborated by transmission electron microscopy images (Figure 5a–d). Figure 5a shows a lattice spacing of 0.24 nm, corresponding to the (111) facet of the FCC Pt structure, while Figure 5b,c reveals two distinct families of particle aggregates. One family was composed of aggregated primary particles (highlighted by a red circle in Figure 5b), while the other consisted of a set of aggregates (blue circle in Figure 5c). The bimodal size distribution of the Pt NP, shown by the NP radius distribution (Figure 5c), was consistent with the hierarchical bimodal families of particles observed by SAXS for the 150AA sample. The TEM images of the 50AA and 100AA samples showed the same characteristics as the 150AA sample, as shown in Figure S4a–d. Figure 5d shows a dark field TEM image, indicating the polycrystalline nature of the aggregates, similar to the Pt NP characterized by Wang et al.^14^ The crystallites presented diameters of around 3–5 nm, in accordance with the *R*_g,p_ values obtained by SAXS.

**Figure 5:**
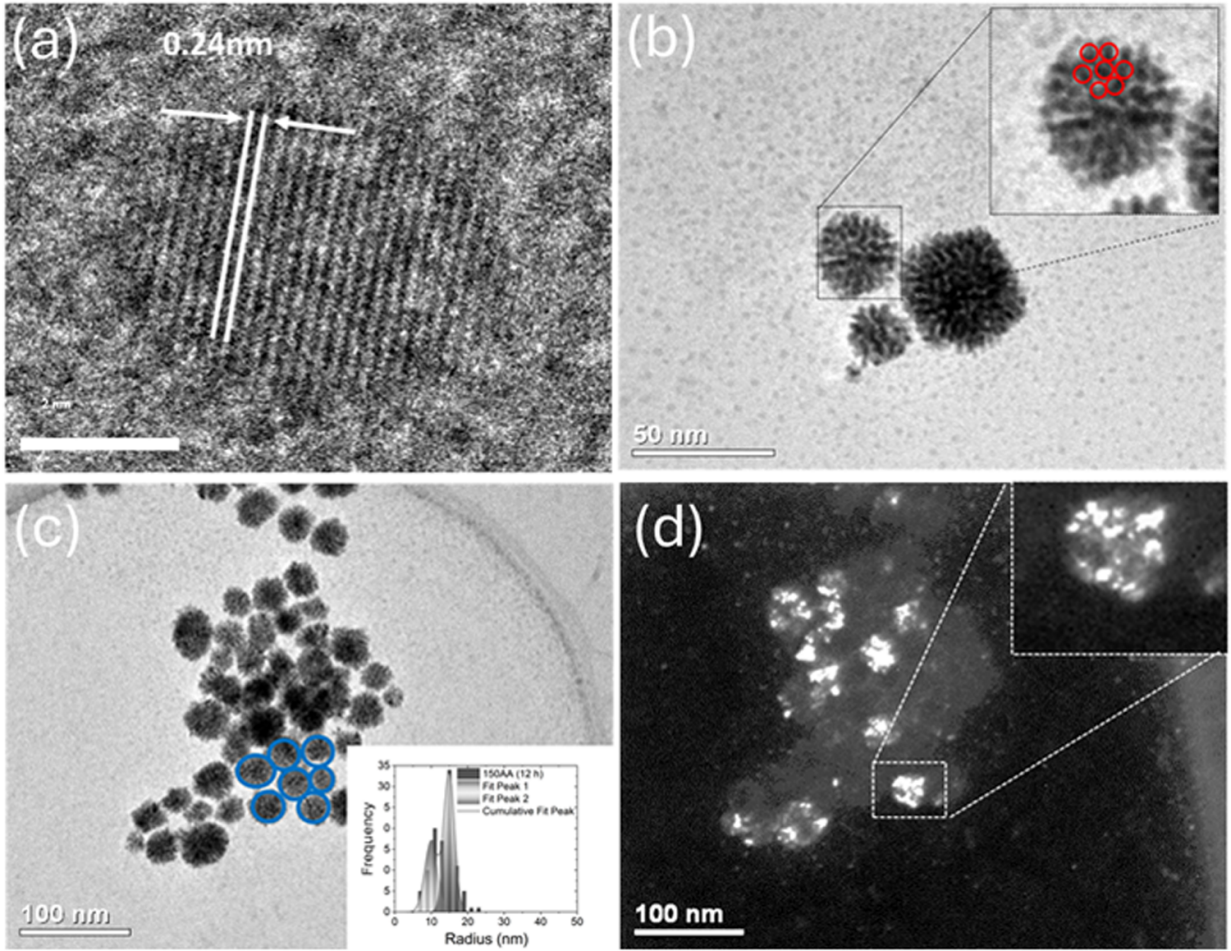
TEM images of the 150AA sample: (a) NP_p_ single crystal with a lattice spacing of 0.24 nm; (b) aggregate, highlighting the neighborhood of primary nanoparticles (family 1); (c) bright-field image, highlighting the neighborhood of the aggregates (family 2), and histogram showing the bimodal size distribution of the Pt NP, consistent with the presence of two families of particles; and (d) dark field image showing the presence of NP_p_ in aggregates.

Based on data obtained from the SAXS, UV–vis, and TEM analyses, as well as previously published work,^51,52^ a four-stage mechanism could be proposed for the formation of the Pt NP, as shown in Figure 6. According to this mechanism, in stage I, nucleation and growth of nanoparticles occurred in two steps, beginning with an induction involving the reduction of Pt atoms followed by the nucleation of crystallized nanoparticles (Figure 6a). In stage II, the system entered the second growth process, after the depletion of monomers caused by the initial nucleation, according to an atom-by-atom addition mechanism. In this process, the single crystalline nanoparticles started to coalesce with each other to form larger nanocrystals, with perfect crystallographic alignment. Additionally, crystal growth with different orientations was observed, indicating prealignment by rotation, as previously suggested by Dachraoui et al.^52^ Stage III involved hierarchical aggregation, with clustering of the NP_p_ nanocrystals to form larger porous structures belonging to families 1 and 2. This occurred in two different steps, IIIa and IIIb, corresponding to initial rapid aggregation, followed by a second stage of slow growth, where aggregation probably continued by the addition of primary nanocrystals of family 1 to the surfaces of the family 2 structures. Finally, in stage IV, the particle aggregates interacted to form more complex structures, creating large aggregates consisting of particles from families 1 and 2.

**Figure 6:**
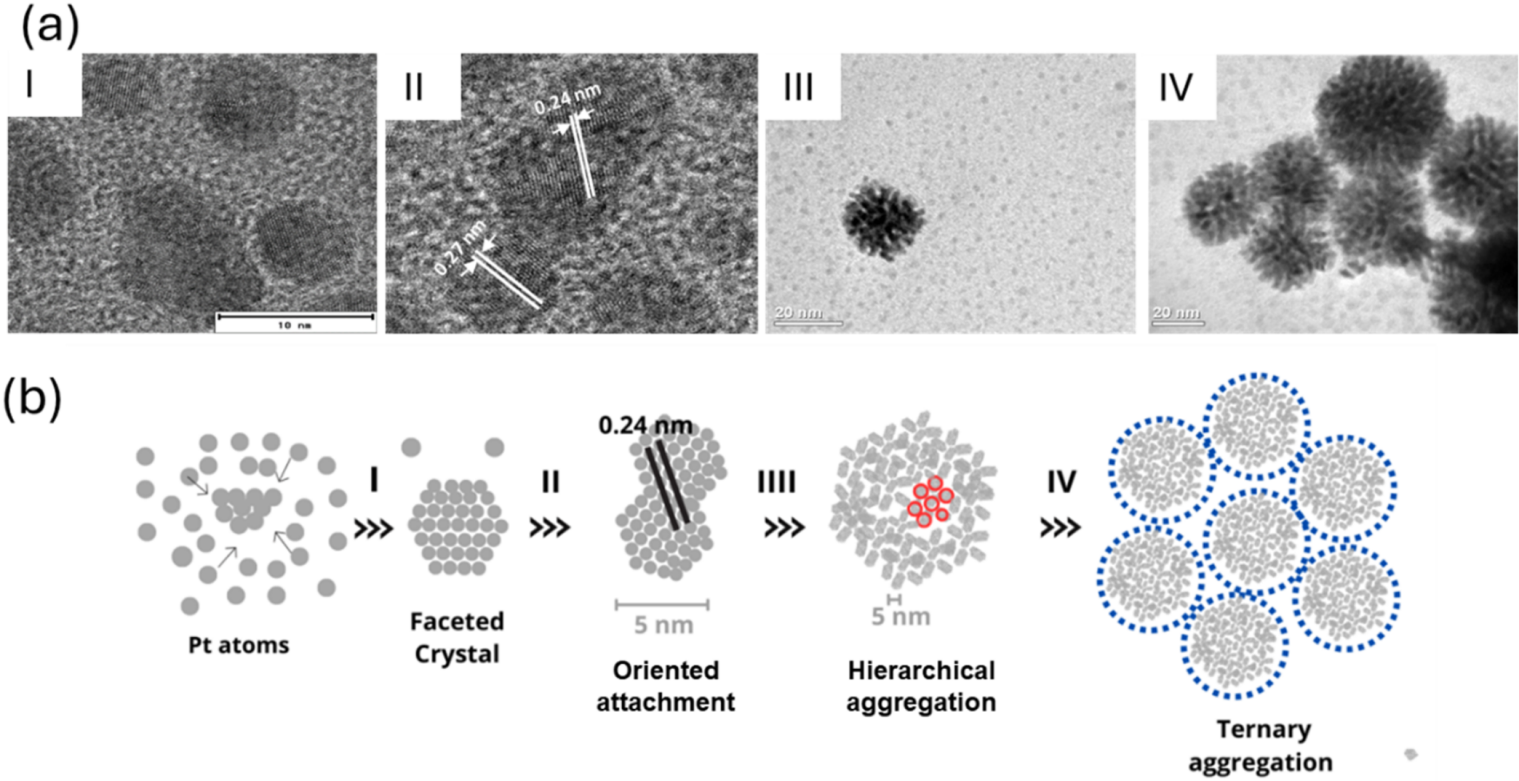
(a) TEM images of particles formed during stages I–IV, and (b) schematic illustration showing the mechanism of formation of platinum aggregates.

Previous studies have demonstrated that AA,^14^ beyond its well-known reducing properties, can influence the final morphology of platinum nanostructures through its oxidation byproducts, which may act as shape-directing agents. In the present work, although the reduction with AA is also believed to govern the fundamental nucleation and anisotropic growth steps, our findings highlight how the presence of TTAB as a stabilizing agent controls the kinetics of aggregation and hierarchical organization of the primary nanoparticles, as will be further discussed alongside with their implication.

### Aggregation Kinetics

The effect of the cationic surfactant TTAB on aggregate growth kinetics was investigated by evaluating the temporal evolution of *N*_p_/*N*_*x*_, using an adjusted time scale in which the initial period (Δ*t*_i_) was subtracted to account exclusively for the aggregation stages (III and IV), during which families 1 and 2 were formed. As shown in Figure 7a–c, *N*_p_/*N*_*x*_ initially increased rapidly, consistent with a burst stage followed by slower growth. Regardless of the TTAB/Pt^2+^ ratio, two linear regions were observed for both nanoparticle families, where the linear coefficients were the rate constants, referred to as *k*_*x*_′ in the first region and *k*_*x*_″ in the second region, where x denotes each nanoparticle family. Notably, the rate constants for both families showed similar trends (*k*_1_′ ≈ *k*_2_′ and *k*_1_″ ≈ *k*_2_″).

**Figure 7:**
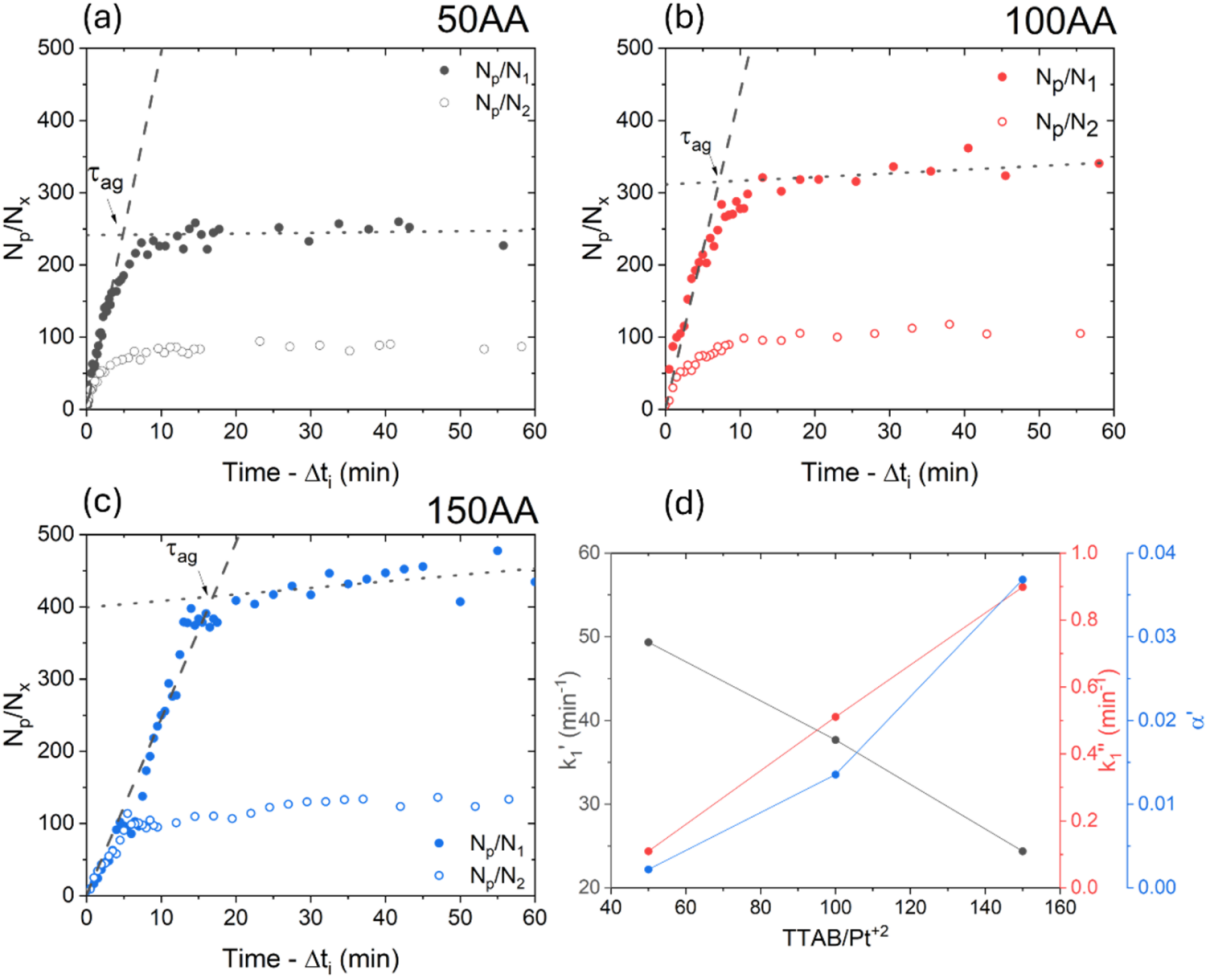
Temporal evolution of *N*_p_/*N*_*x*_ for each family of particles, for samples (a) 50AA, (b) 100AA, and (c) 150AA; (d) absolute rate constants for volume growth, *k*_1_′ and *k*_1_″, and the collision efficiency, α′, after τ_ag_, according to the TTAB/Pt^2+^ ratio.

The presence of two linear regimes indicated that the efficiency of collision (α′), or attachment of NP_p_, described by Fuchs,^53^ changed after τ_ag_, the time when the intersection between the two linear regimes occurred. This reduction in the growth rate could be explained by the effect of repulsive colloidal interactions on perikinetic aggregation.^45^ The attachment efficiency is a fundamental parameter that quantifies how readily particles aggregate upon collision, serving as an inverse measure of system stability and helping to distinguish between different aggregation regimes.^45,54^ The normalization of the observed aggregation rate constants provides a standardized framework to evaluate and compare aggregation behaviors under different conditions. Here, α′ was calculated by normalizing *k*_1_″ by *k*_1_′ (eq 9), giving the collision efficiency after τ_ag_. The evolution of α′ as a function of the TTAB/Pt^2+^ ratio is shown in Figure 7d, together with the values of *k*_1_′ and *k*_1_″.

9 $$\alpha^{\prime }=\frac{{k_{1}}^{\prime \prime }}{{k_{1}}^{\prime }}$$

The results indicated that during the slower regime, an increase in the surfactant amount led to an increase of *k*_1_″ and higher collision efficiency, which may have been due to the higher amount of free NP_p_ that had not aggregated during the fast regime. It could be assumed that during the fast growth period, α′ should be higher and much closer to 1, as expected for a diffusion-limited aggregation growth process,^54^ while α′ should decrease with the amount of TTAB, at the same rate as *k*_1_′(TTAB/Pt^2+^). Following τ_ag_, the aggregation process became less efficient due to a transition to a reaction-limited regime. This behavior reflects the competing effects of universal Coulombic attraction and steric repulsion arising from surface coating layers, which significantly influenced the kinetics of aggregate growth.^54^

Adsorbed or covalently bonded surfactants can prevent aggregation and enhance the stability of NP dispersions by increasing the surface charge and electrostatic repulsion or by reducing the interfacial energy between particle and solvent. For example, compared with uncoated silver NP, the critical coagulation concentrations of poly­(vinylpyrrolidone)-silver NP (PVP-AgNP) and citrate-AgNP were found to be more than 4-fold and 2-fold higher, respectively.^55^ Surfactant chain length, molecular weight, and types of head groups, together with the affinity of coating molecules for the particle surface, repulsion from neighboring molecules, loss of chain entropy upon adsorption, and nonspecific dipole interactions between the macromolecule, the solvent, and the surface can significantly affect the adsorbed surfactant mass and layer conformation, consequently influencing the ability of a surfactant to stabilize NP against aggregation.^56,57^ In the present system, after formation of the NP_p_, the surface of the metal should be positively charged, given its isoelectric pH (around 3)^58,59^ and the pH of the reaction media (around 2.3 and 2.5), causing the absorption of halide^40^ at the surface of the metal. Negatively charged halides should provide an attractive force for formation of a bilayer of the positively charged surfactant (TTA^+^) around the primary nanoparticle,^15^ which could provide electrosteric stabilization of the NP.^54^

The effect of TTAB on the synthesis could also be seen from several structural features. As shown in Figure 8a, the primary nanoparticle gyration radius and polydispersity of the NP_p_ (σ_p_) both decreased with increasing TTAB concentration, as observed in the TEM images (Figure S5a–f), which was consistent with the behavior typically expected for a capping agent.^22^ However, this effect appeared to contradict the aggregation behavior predicted by DLVO theory, which suggests that a smaller nanoparticle size should reduce the energy barrier for aggregation.^54^ TTAB is in excess^60^ in the system proposed in this study, being up to 150 molecules of TTAB per atom Pt^2+^, which could explain the behavior for aggregation rate *k*_1_′ and Δ*t*_i_, as a function of TTAB (Figure 8b), suggesting that the activation barrier increased, probably because aggregation was hindered by a more effective electrosteric barrier.

**Figure 8:**
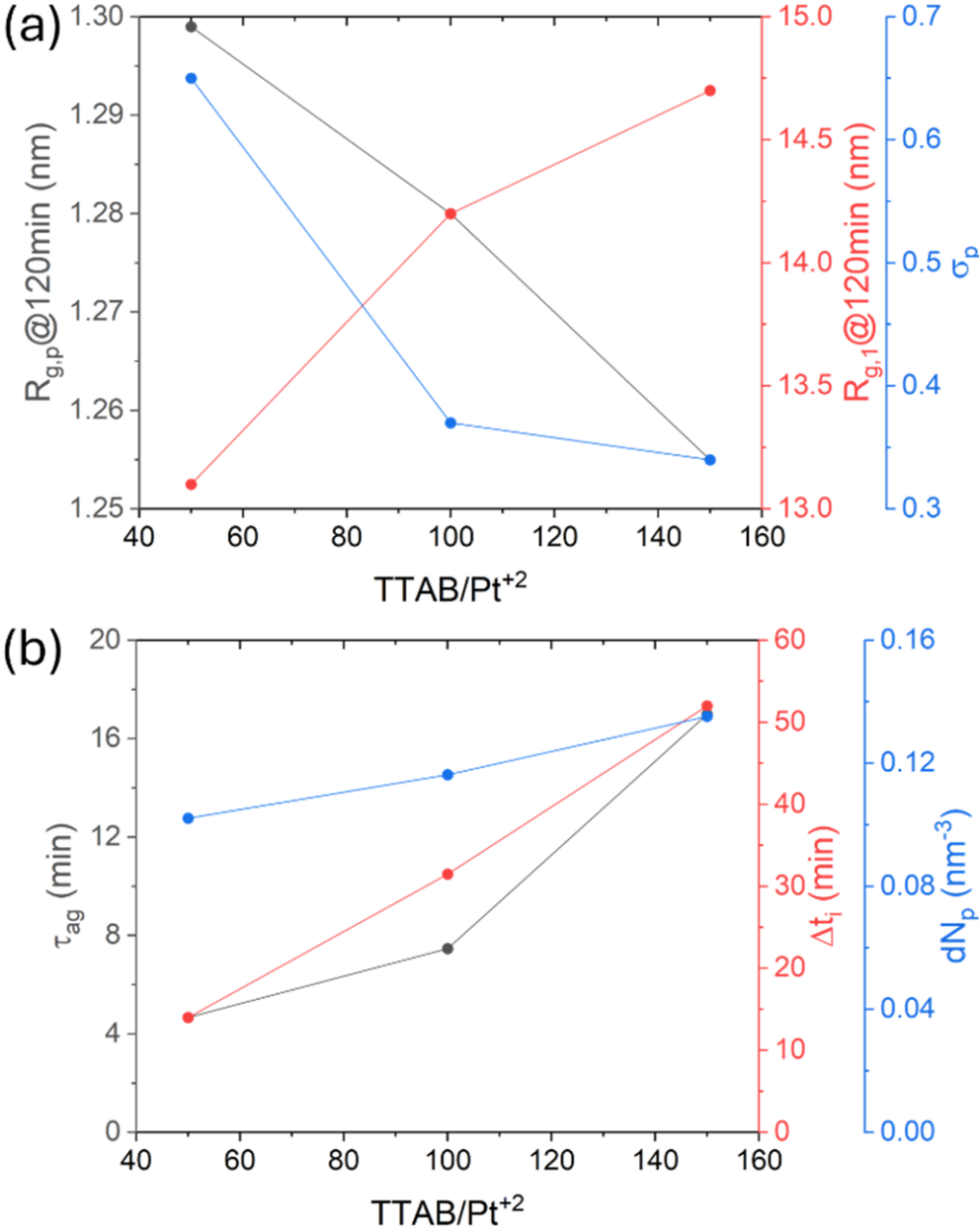
(a) Effect of the TTAB/Pt^2+^ ratio on the polydispersity of the primary nanoparticles (σ_p_) and the gyration ratios for the primary particles (*R*_g,p_) and aggregates (*R*_g,1_) measured at the end of the reaction (120 min). (b) Effect of the TTAB/Pt^2+^ ratio on the evolution of incubation time (Δ*t*_i_), aggregate porosity (d*N*_p_), and time for crossover between aggregation regimes (τ_ag_).

It was also observed that the calculated density of NP_p_ (eq 8) within aggregates from family 1 (d*N*_p_), shown in Figure 8b, increased with the amount of surfactant, as expected because the reduced dispersity and smaller particle size enabled more efficient packing. This suggested that aggregate porosity decreased with an increase in the surfactant amount. Another notable trend, shown in Figure 8b, was that τ_ag_ increased with the TTAB concentration, indicating that the initial aggregation step was extended with the increase of TTAB, providing further insight into the role of TTAB during the aggregation process. Additionally, as shown in Figure 8a, the gyration radius for family 1 (*R*_g,1_) at the end of the reaction (120 min) was inversely proportional to that of *R*_g,p_ at 120 min, consistent with a more controlled growth process.

In contrast to previous studies^14,41^ that focused primarily on the final morphology of Pt nanoparticles formed in the presence of TTAB or under surfactant-free conditions, our results provide mechanistic insights into how TTAB actively modulates the nucleation and aggregation processes. We observed that increasing the TTAB concentration led to a reduction in both the primary particle size (*R*_g,p_) and polydispersity (σ_p_), consistent with the expected capping behavior of surfactants. However, despite smaller particle sizes theoretically favoring aggregation by DLVO theory, our data showed that aggregation became slower with higher TTAB levels. This behavior is consistent with the presence of a more effective electrosteric barrier, likely due to the large excess of TTAB relative to that of Pt^2+^. Furthermore, the increased density of primary nanoparticles within aggregates (d*N*_p_) and the inverse relationship between the final aggregate size (*R*_g,1_) and primary particle size indicate that TTAB not only controls primary particle formation, but also affects packing efficiency and aggregate porosity, which could ultimately impact in performance in different application, such as catalysis.^7^ These findings expand upon previous reports by showing that TTAB plays a dual role: not only influencing final shape and size as previously suggested but also modulating reaction kinetics and aggregation pathways, offering a more comprehensive understanding of surfactant-mediated nanoparticle formation.

### Role of TTAB in the Overall Growth Mechanism

To further understand the role of TTAB in the kinetics of Pt nanoparticle formation and growth during synthesis of the 50AA, 100AA, and 150AA samples, SAXS analyses were performed for the solutions in which the reaction occurred, before and immediately after the addition of AA to the solution with 100 mM TTAB (Figure 9). The addition of Pt^2+^ caused a reduction in the scattering intensity produced by the TTAB micellar structures in the solution, which could be explained by a decrease of the electronic density contrast due to the presence of the platinum salt in the solution matrix. From this, it could be concluded that before the addition of AA, the ions of the platinum salt were in solution, outside the structure of the TTAB micelles. Hence, after the formation of the primary nanoparticles, an attractive force, such as the surface charges that led to the dislocation of TTA^+^ and Br^–^, initially acted to prevent aggregation. The scattering intensity around the correlation peak (*q* < 1 nm^–1^), associated with the average intermicellar distance, increases linearly with increasing TTAB concentration (Figure S6). This behavior cannot be attributed solely to contrast variation and instead suggests a physical change in micelle–micelle interactions. As the concentration of TTAB increases, so does the amount of bromide ions in the solution, effectively increasing the ionic strength. This leads to a reduction in the Debye screening length, κ^–1^, which in turn decreases the range of electrostatic repulsion between micelles.^61^ The weakening of this repulsion allows micelles to approach more closely, resulting in a reduced intermicellar distance, as indicated by the reduction in correlation distance indicated in Figure S6. This effect is reflected in the shift and broadening of the correlation peaks in the scattering curves. The more compact micellar arrangement may also contribute to the slower exchange kinetics observed at higher TTAB concentrations as diffusion and rearrangement become more restricted in a crowded environment.

**Figure 9:**
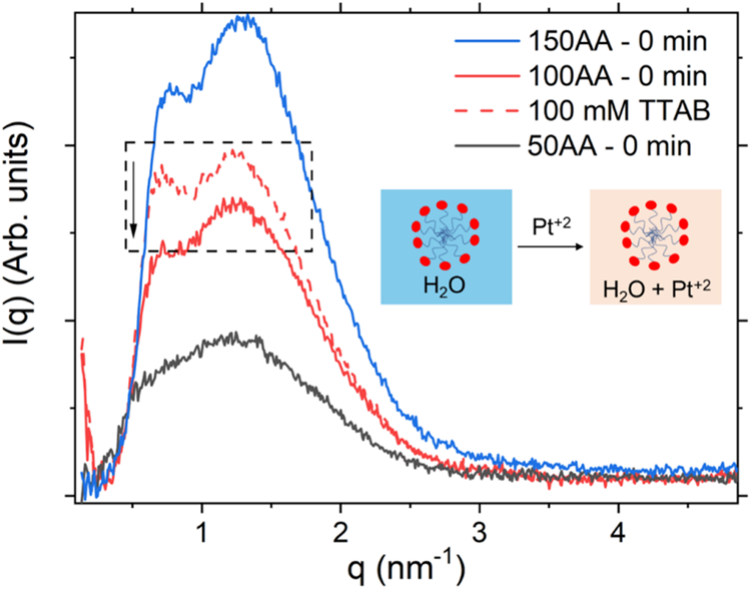
SAXS curves for the surfactant solution before (dashed red line) and after (solid red line) the addition of the Pt^2+^ solution, for the sample with TTAB/Pt^2+^ = 100. Also shown are the curves for the 50AA and 150AA samples at *t* = 0.

Based on these findings, together with the results discussed in the previous sections, an overall mechanism could be proposed for the formation and growth of platinum aggregates. In the first stage of the synthesis, there was an incubation period during which platinum NP_p_ was nucleated, as indicated by the UV–vis analyses. The presence of TTAB in the reaction medium prevented the rapid aggregation of the primary particles, with TTAB acting to stabilize the nanoparticles. This suggested that during the formation of the first nuclei, a driving force displaced the TTAB molecules from the micelles to the Pt surface. As the amount of TTAB in the medium increased, stabilization of the Pt nanoparticles became more effective, delaying the aggregative process. The driving force for this later process was the reduction of the excess surface energy of NP_p_, since smaller nanoparticles were associated with higher excess surface energy. As the number of NP_p_ increased, the balance of opposing forces was shifted, favoring a reduction in the excess surface energy due to the formation of aggregates. The absolute aggregation rate for the fast aggregation window, prior to τ_ag_, was inversely related to the TTAB/Pt^2+^ ratio, indicating that the difference between the attractive and repulsive forces driving the aggregation process tended to decrease with an increase in surfactant content. Additionally, after τ_ag_, a second slower aggregation process took place. This process was proportional to the amount of TTAB and can be attributed to both an excess of nonaggregated NP_p_ and a less densely packed micellar environment.

## Conclusions

This study provides a detailed mechanistic understanding of the formation and growth of platinum nanoparticles synthesized by reduction with ascorbic acid and stabilized by the cationic surfactant TTAB. Based on combined SAXS, UV–vis, XAS, and TEM analyses, a four-stage growth mechanism was proposed, revealing that Pt nanoparticle evolution is governed by a dynamic interplay of attractive and repulsive forces with TTAB playing a key role in modulating aggregation kinetics. The surfactant was shown to delay aggregation by promoting electrosteric stabilization, particularly at higher concentrations, resulting in smaller and more monodisperse primary particles, denser aggregate packing, and reduced aggregate porosity. These findings deviate from classical DLVO predictions and extend previous studies on ascorbic acid, which primarily focused on its role as a reducing agent and morphology-directing species through oxidation byproducts. While the reduction pathway remains central to determining particle shape, our results demonstrate that TTAB introduces an additional and previously underexplored level of control over the aggregation and hierarchical assembly processes. These insights underscore the critical importance of surfactant concentration as a tunable parameter in nanoparticle design, with implications for tailoring particle size, structure, and porosity in applications ranging from catalysis to nanomaterials engineering.

Importantly, the use of *in situ*, time-resolved SAXS was instrumental in tracking the evolution of key parameters, such as particle size and aggregate formation dynamics. This approach enabled the identification of subtle kinetic transitions throughout the aggregation process—including delayed nucleation, changes in packing efficiency, and modulation of aggregate porosity, that could not be readily observed with conventional *ex situ* techniques. These findings not only provide a more complete picture of the Pt NP formation mechanism but also demonstrate the value of *in situ* SAXS for guiding the rational design of nanoparticle-based materials.

## Supplementary Material



## Notes

Complementary information on the experimental setup (Figure S1) and of the fitting procedure of SAXS curves (Figure S2). Time-resolved UV–vis data showing the absorption band shift, and EXAFS spectra (Figure S3). TEM images of primary nanoparticles (Figure S4 and S5). Scattering intensity around the correlation peak associated with the average intermicellar distance (Figure S6) (PDF)

## References

[1] Shi Y., Lyu Z., Zhao M., Chen R., Nguyen Q. N., Xia Y. (2021). Noble-Metal
Nanocrystals with Controlled Shapes for Catalytic and Electrocatalytic
Applications. Chem. Rev..

[2] Kim H., Yoo T. Y., Bootharaju M. S., Kim J. H., Chung D. Y., Hyeon T. (2022). Noble Metal-Based Multimetallic
Nanoparticles for Electrocatalytic
Applications. Adv. Sci..

[3] Lord R. W., Holder C. F., Fenton J. L., Schaak R. E. (2019). Seeded Growth of
Metal Nitrides on Noble-Metal Nanoparticles To Form Complex Nanoscale
Heterostructures. Chem. Mater..

[4] Xu G. R., Wang B., Zhu J. Y., Liu F. Y., Chen Y., Zeng J. H., Jiang J. X., Liu Z. H., Tang Y. W., Lee J. M. (2016). Morphological
and Interfacial Control of Platinum Nanostructures
for Electrocatalytic Oxygen Reduction. ACS Catal..

[5] Watt J., Cheong S., Toney M. F., Ingham B., Cookson J., Bishop P. T., Tilley R. D. (2010). Ultrafast Growth of Highly Branched
Palladium Nanostructures for Catalysis. ACS
Nano.

[6] Wang L., Holewinski A., Wang C. (2018). Prospects of Platinum-Based Nanostructures
for the Electrocatalytic Reduction of Oxygen. ACS Catal..

[7] Song Y., Yang Y., Medforth C. J., Pereira E., Singh A. K., Xu H., Jiang Y., Brinker C. J., van Swol F., Shelnutt J. A. (2004). Controlled
Synthesis of 2-D and 3-D Dendritic Platinum Nanostructures. J. Am. Chem. Soc..

[8] Song Y., Steen W. A., Peña D., Jiang Y. B., Medforth C. J., Huo Q., Pincus J. L., Qiu Y., Sasaki D. Y., Miller J. E., Shelnutt J. A. (2006). Foamlike Nanostructures
Created from Dendritic Platinum
Sheets on Liposomes. Chem. Mater..

[9] Pan Y., Blum A. S., Mauzeroll J. (2021). Tunable Assembly
of Protein Enables
Fabrication of Platinum Nanostructures with Different Catalytic Activity. ACS Appl. Mater. Interfaces.

[10] Gautam S., Chugh S., Gates B. D. (2023). Evaluating
the Effects of Surfactant
Templates on the Electrocatalytic Activity and Durability of Multifaceted
Platinum Nanostructures. ACS Appl. Energy Mater..

[11] Kalekar A. M., Sharma K. K. K., Lehoux A., Audonnet F., Remita H., Saha A., Sharma G. K. (2013). Investigation into the Catalytic
Activity of Porous Platinum Nanostructures. Langmuir.

[12] Wang L., Wang H., Nemoto Y., Yamauchi Y. (2010). Rapid and Efficient
Synthesis of Platinum Nanodendrites with High Surface Area by Chemical
Reduction with Formic Acid. Chem. Mater..

[13] Chang S. L.
Y., Barnard A. S., Dwyer C., Hansen T. W., Wagner J. B., Dunin-Borkowski R. E., Weyland M., Konishi H., Xu H. (2012). Stability
of Porous Platinum Nanoparticles: Combined In Situ TEM and Theoretical
Study. J. Phys. Chem. Lett..

[14] Wang L., Hu C., Nemoto Y., Tateyama Y., Yamauchi Y. (2010). On the Role of Ascorbic
Acid in the Synthesis of Single-Crystal Hyperbranched Platinum Nanostructures. Cryst. Growth Des..

[15] Joo S. H., Park J. Y., Tsung C.-K., Yamada Y., Yang P., Somorjai G. A. (2009). Thermally Stable
Pt/Mesoporous Silica Core–Shell
Nanocatalysts for High-Temperature Reactions. Nat. Mater..

[16] Xia Y., Xiong Y., Lim B., Skrabalak S. E. (2009). Shape-Controlled
Synthesis of Metal Nanocrystals: Simple Chemistry Meets Complex Physics?. Angew. Chem., Int. Ed..

[17] Leong G. J., Schulze M. C., Strand M. B., Maloney D., Frisco S. L., Dinh H. N., Pivovar B., Richards R. M. (2014). Shape-Directed Platinum
Nanoparticle Synthesis: Nanoscale Design of Novel Catalysts. Appl. Organomet. Chem..

[18] Satyavolu N. S. R., Tan L. H., Lu Y. (2016). DNA-Mediated
Morphological Control
of Pd–Au Bimetallic Nanoparticles. J.
Am. Chem. Soc..

[19] Quinson J., Jensen K. M. Ø. (2020). From Platinum
Atoms in Molecules to Colloidal Nanoparticles:
A Review on Reduction, Nucleation and Growth Mechanisms. Adv. Colloid Interface Sci..

[20] Borodko Y., Humphrey S. M., Tilley T. D., Frei H., Somorjai G. A. (2007). Charge-Transfer
Interaction of Poly­(Vinylpyrrolidone) with Platinum and Rhodium Nanoparticles. J. Phys. Chem. C.

[21] Luty-Błocho M. (2019). The Influence
of Steric Stabilization on Process of Au, Pt Nanoparticles Formation. Arch. Metall. Mater..

[22] Song T., Gao F., Guo S., Zhang Y., Li S., You H., Du Y. (2021). A Review of
the Role and Mechanism of Surfactants in the Morphology
Control of Metal Nanoparticles. Nanoscale.

[23] Javadian S., Nasiri F., Heydari A., Yousefi A., Shahir A. A. (2014). Modifying
Effect of Imidazolium-Based Ionic Liquids on Surface Activity and
Self-Assembled Nanostructures of Sodium Dodecyl Sulfate. J. Phys. Chem. B.

[24] Marcolongo J. P., Mirenda M. (2011). Thermodynamics of Sodium
Dodecyl Sulfate (SDS) Micellization:
An Undergraduate Laboratory Experiment. J. Chem.
Educ..

[25] Xu J., Mueller R., Hazelbaker E., Zhao Y., Bonzongo J. C. J., Clar J. G., Vasenkov S., Ziegler K. J. (2017). Strongly Bound Sodium
Dodecyl Sulfate Surrounding Single-Wall Carbon Nanotubes. Langmuir.

[26] Huynh K. A., Chen K. L. (2011). Aggregation Kinetics
of Citrate and Polyvinylpyrrolidone
Coated Silver Nanoparticles in Monovalent and Divalent Electrolyte
Solutions. Environ. Sci. Technol..

[27] Koczkur K.
M., Mourdikoudis S., Polavarapu L., Skrabalak S. E. (2015). Polyvinylpyrrolidone
(PVP) in Nanoparticle Synthesis. Dalton Trans..

[28] Luo S., Shen P. K. (2017). Concave Platinum-Copper
Octopod Nanoframes Bounded
with Multiple High-Index Facets for Efficient Electrooxidation Catalysis. ACS Nano.

[29] Zhang Y., Grass M. E., Kuhn J. N., Tao F., Habas S. E., Huang W., Yang P., Somorjai G. A. (2008). Highly
Selective
Synthesis of Catalytically Active Monodisperse Rhodium Nanocubes. J. Am. Chem. Soc..

[30] Yan X., Yu S., Tang Y., Sun D., Xu L., Xue C. (2018). Triangular
AgAu@Pt Core-Shell Nanoframes with a Dendritic Pt Shell and Enhanced
Electrocatalytic Performance toward the Methanol Oxidation Reaction. Nanoscale.

[31] Finney E. E., Finke R. G. (2008). Nanocluster Nucleation
and Growth Kinetic and Mechanistic
Studies: A Review Emphasizing Transition-Metal Nanoclusters. J. Colloid Interface Sci..

[32] Angermund K., Bühl M., Endruschat U., Mauschick F. T., Mörtel R., Mynott R., Tesche B., Waldöfner N., Bönnemann H., Köhl G., Modrow H., Hormes J., Dinjus E., Gassner F., Haubold H.-G., Vad T., Kaupp M. (2003). In Situ Study on the Wet Chemical Synthesis of Nanoscopic Pt Colloids
by “Reductive Stabilization.”. J. Phys. Chem. B.

[33] Angermund K., Bühl M., Dinjus E., Endruschat U., Gassner F., Haubold H.-G., Hormes J., Köhl G., Mauschick F. T., Modrow H., Mörtel R., Mynott R., Tesche B., Vad T., Waldöfner N., Bönnemann H. (2002). Nanoscopic
Pt Colloids in the “Embryonic State.”. Angew. Chem., Int. Ed..

[34] Borodko Y., Ercius P., Zherebetskyy D., Wang Y., Sun Y., Somorjai G. (2013). From Single Atoms to
Nanocrystals: Photoreduction of
[PtCl 6 ] 2– in Aqueous and Tetrahydrofuran Solutions of PVP. J. Phys. Chem. C.

[35] Borodko Y., Ercius P., Pushkarev V., Thompson C., Somorjai G. (2012). From Single
Pt Atoms to Pt Nanocrystals: Photoreduction of Pt 2+ Inside of a PAMAM
Dendrimer. J. Phys. Chem. Lett..

[36] Lagrow A. P., Ingham B., Toney M. F., Tilley R. D. (2013). Effect of Surfactant
Concentration and Aggregation on the Growth Kinetics of Nickel Nanoparticles. J. Phys. Chem. C.

[37] Richards V. N., Shields S. P., Buhro W. E. (2011). Nucleation
Control in the Aggregative
Growth of Bismuth Nanocrystals. Chem. Mater..

[38] Shields S.
P., Richards V. N., Buhro W. E. (2010). Nucleation Control of Size and Dispersity
in Aggregative Nanoparticle Growth. A Study of the Coarsening Kinetics
of Thiolate-Capped Gold Nanocrystals. Chem.
Mater..

[39] Mourdikoudis, S. ; Liz-marza, L. M. 2013_Oleylamine in Nanoparticle Synthesis_Mourdikoudis, Liz-Marza_Unknown.Pdf. 2013.

[40] Lohse S. E., Burrows N. D., Scarabelli L., Liz-Marzán L. M., Murphy C. J. (2014). Anisotropic Noble Metal Nanocrystal
Growth: The Role
of Halides. Chem. Mater..

[41] Seo J., Lee S., Koo B., Jung W. (2018). Controlling the Size of Pt Nanoparticles
with a Cationic Surfactant, CnTABr. CrystEngComm.

[42] Li T., Senesi A. J., Lee B. (2016). Small Angle
X-Ray Scattering for
Nanoparticle Research. Chem. Rev..

[43] Beaucage G. (1995). Approximations
Leading to a Unified Exponential/Power-Law Approach to Small-Angle
Scattering. J. Appl. Crystallogr..

[44] Santilli C. V., Sarmento V. H. V., Dahmouche K., Pulcinelli S. H., Craievich A. F. (2009). Effects of Synthesis Conditions on
the Nanostructure
of Hybrid Sols Produced by the Hydrolytic Condensation of (3-Methacryloxypropyl)­Trimethoxysilane. J. Phys. Chem. C.

[45] Elimelech, M. ; Jia, X. ; Gregory, J. ; Williams, R. Modelling of Aggregation Processes. In Particle Deposition & Aggregation; Elsevier, 1998; Chapter 6.

[46] Ravel B., Newville M. (2005). ATHENA, ARTEMIS, HEPHAESTUS: data analysis for X-ray
absorption spectroscopy using IFEFFIT. Synchrotron
Radiat..

[47] Lutz-Bueno V., Liebi M., Kohlbrecher J., Fischer P. (2017). Intermicellar Interactions
and the Viscoelasticity of Surfactant Solutions: Complementary Use
of SANS and SAXS. Langmuir.

[48] Lee H., Habas S. E., Kweskin S., Butcher D., Somorjai G. A., Yang P. (2006). Morphological Control
of Catalytically Active Platinum Nanocrystals. Angew. Chem., Int. Ed..

[49] Chen S., Yang Q., Wang H., Zhang S., Li J., Wang Y., Chu W., Ye Q., Song L. (2015). Initial Reaction
Mechanism of Platinum Nanoparticle in Methanol–Water System
and the Anomalous Catalytic Effect of Water. Nano Lett..

[50] Craievich, A. F. Small-Angle X-Ray Scattering by Nanostructured Materials. In Handbook of Sol-Gel Science and Technology; Klein, L. ; Aparicio, M. ; Jitianu, A. , Eds.; Springer International Publishing: Cham, 2018; pp 1185–1230.

[51] Kim J., Kang D., Kang S., Kim B. H., Park J. (2022). Coalescence
Dynamics of Platinum Group Metal Nanoparticles Revealed by Liquid-Phase
Transmission Electron Microscopy. iScience.

[52] Dachraoui W., Henninen T. R., Keller D., Erni R. (2021). Multi-Step Atomic Mechanism
of Platinum Nanocrystals Nucleation and Growth Revealed by in-Situ
Liquid Cell STEM. Sci. Rep..

[53] Fuchs N. (1934). Theory of
Coagulation. Z. Phys. Chem., Abt. B.

[54] Zhang W. (2014). Nanoparticle
Aggregation: Principles and Modeling. Adv. Exp.
Med. Biol..

[55] Kittler S., Greulich C., Köller M., Epple M. (2009). Synthesis of PVP-Coated
Silver Nanoparticles and Their Biological Activity towards Human Mesenchymal
Stem Cells. Materwiss. Werksttech..

[56] Vaisman L., Wagner H. D., Marom G. (2006). The Role of
Surfactants in Dispersion
of Carbon Nanotubes. Adv. Colloid Interface
Sci..

[57] Li X., Lenhart J. J., Walker H. W. (2012). Aggregation
Kinetics and Dissolution
of Coated Silver Nanoparticles. Langmuir.

[58] Kallay N., Torbic Z., Golic M., Matijevic E. (1991). Determination
of the Isoelectric Points of Several Metals by an Adhesion Method. J. Phys. Chem. A.

[59] Ishida K., Tachibana M., Wada Y., Ota N., Aizawa M. (2017). Formation
of Platinum Nanoparticle Colloidal Solution by Gamma-Ray Irradiation. J. Nucl. Sci. Technol..

[60] Alargova R. G., Kochijashky I. I., Sierra M. L., Zana R. (1998). Micelle Aggregation
Numbers of Surfactants in Aqueous Solutions: A Comparison between
the Results from Steady-State and Time-Resolved Fluorescence Quenching. Langmuir.

[61] Shukla A., Rehage H. (2008). Zeta Potentials and
Debye Screening Lengths of Aqueous,
Viscoelastic Surfactant Solutions (Cetyltrimethylammonium Bromide/Sodium
Salicylate System). Langmuir.

