# Supplementary material for: Tuning the Formation and Growth of Platinum Nanoparticles Using Surfactant: In Situ SAXS Study of the Aggregative Growth Mechanism
Source: ACS Appl Mater Interfaces. 2025 Jul 2;17(28):41237–48. doi: 10.1021/acsami.5c05268 (PMC12278246; doi:10.1021/acsami.5c05268)
Supplement: Supplementary file 1 [file am5c05268_si_001.pdf]

Supporting Information

Tuning the Formation and Growth of Platinum Nanoparticles Using Surfactant: In Situ SAXS Study of the Aggregative Growth Mechanism

Rodolfo Fini[a], Marina Magnani[a], Celso Valentim Santilli[a], Sandra H. Pulcinelli[a*]

[a] Institute of Chemistry, São Paulo State University (UNESP), Araraquara, SP, 14800-060, Brazil

[*] Corresponding author e-mail: sandra.h.pulcinelli@unesp.br

Rodolfo Fini ORCID: 0000-0002-4460-8208

Marina Magnani ORCID: 0000-0002-2574-786X

Celso V. Santilli ORCID: 0000-0002-8356-8093

Sandra H. Pulcinelli ORCID: 0000-0003-0783-7463

EXPERIMENTAL SETUP FOR *IN-SITU* SAXS AND UV-VIS ANALYSES

Figure S1 illustrates the experimental setup for in situ data acquisition using the UV-Vis and SAXS techniques.

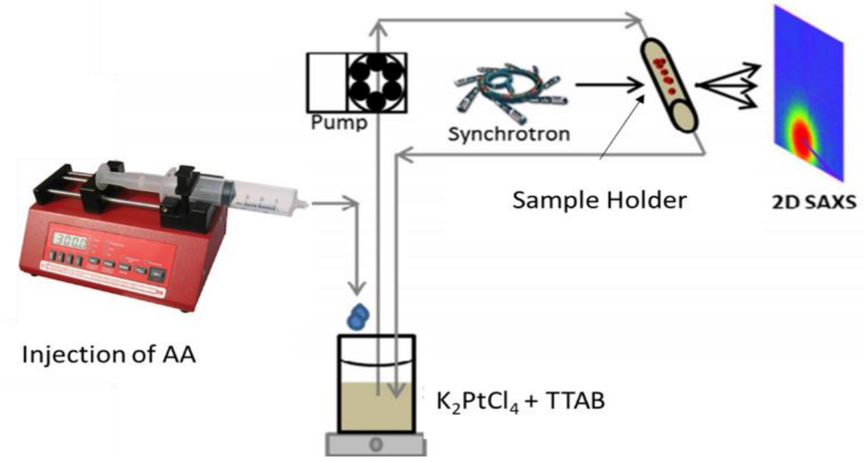

Figure. S1. Schematic representation of the in situ SAXS measurements performed at the SAXS1 beamline of LNLS.

RESULTS

Parameters from Eq. 1 and Eq. 4-6 (mentioned in the original paper) were obtained by fitting the SAXS data in which I(q) was subtracted by I(q) at t = 0 min to disregard the contribution of the TTAB micelles for samples containing TTAB. Figure S2a-c illustrates three representative stages of the 150AA synthesis to exemplify the data processing procedure. Figure S2a shows the SAXS curve at 57 min, which corresponds to the first time point where information from both the nanoparticle family (Family NP) and the primary nanoparticles becomes accessible. At this stage, a two-level scattering system is observed. Prior to this time point, the contribution from TTAB was relatively high, resulting in noisy data that hindered reliable fitting. Figure S2b presents the SAXS profile at 65 min, when the correlation peak associated with the primary nanoparticles reaches its maximum (rendering a packing factor around 5.9, characteristic of HCP structures). At this stage, both nanoparticle families can be clearly identified, characterizing a three-level scattering system. Finally, Figure S2c displays the SAXS curve at the latest time point of the synthesis. Compared to the curve at 65 min, both Guinier regions corresponding to Families 1 and 2 are shifted towards lower q-values, indicating particles growth over time. Figure S2d represent the three-level system observed with SAXS data, obtained with TEM images.

In Figures S2a–c, we also present the Guinier fits for each structural level, along with the Beaucage unified fit applied specifically to the primary nanoparticles and to the full scattering

profile. During the fitting procedure, the Porod exponent was fixed at 4 to reflect smooth interfaces, while the remaining parameters were allowed to vary within physically meaningful ranges. Data analysis for primary level was fully evaluated using Igor Pro v. 9.05[1] with Irena Package v. 1.16[2]. To evaluate particles from families 1 and 2 we have used OriginPro 2024[3] after confirmed a good agreement between radius of gyration and I_0 calculated by both unified model and Guinier equation. In the Figure S2 we present the unified fit model obtained using radius of gyration and I_0 parameters obtained by Guinier approach. The difference between the curves derived from Guinier and unified fit using Beaucage (Eq. 4), observed at q < 0.5nm, gives an idea of the repulsive interaction between the correlated primary particles.

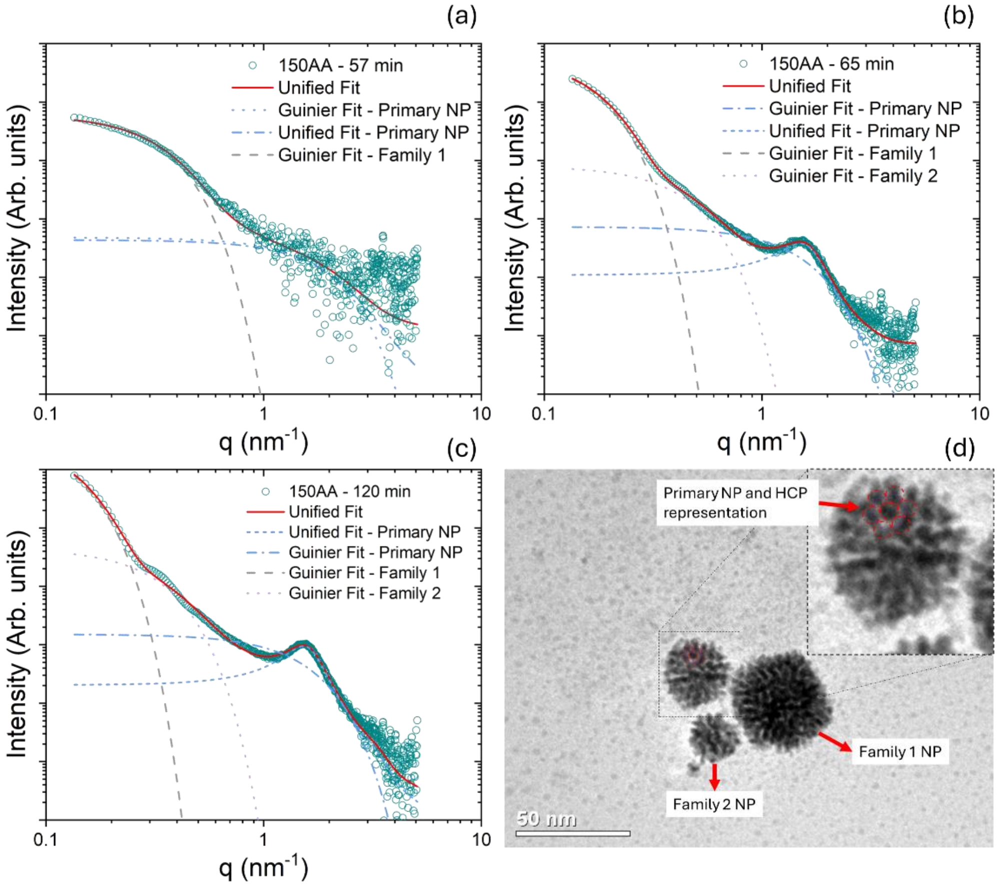

Figure. S2. SAXS curves and corresponding fits at different time points of the 150AA synthesis: (a) 57 min, (b) 65 min, and (c) final time point. Guinier fits for each level and Beaucage unified fits for the primary nanoparticles and full profiles are shown. (d) Schematic three-level scattering system obtained from TEM.

Figure S3a presents the in situ UV-Vis data, where an initial shift of the band position from approximately 390 to 420 nm can be seen. This transition is more clearly shown in Figure S3b, which compares the UV-Vis spectra of a K_2PtCl_4 solution and K_2PtCl_4+ TTAB. This shift was related to a change in the first coordination shell of the platinum ion, caused by the exchange from chloride to bromide ligands. This change was further confirmed by the EXAFS data,

shown in Figure S3c-d. The EXAFS data fitting is shown in Table S1, revealing a shift in the Pt^{2+} first coordination shell distance from 2.30 to 2.43 Å, attributed to the larger size of bromide than chloride atoms. Table S1 also indicates that the coordination number in the first shell of the platinum nanoparticles was less than 12, probably due to the reduced nanoparticle size, compared to platinum foil.

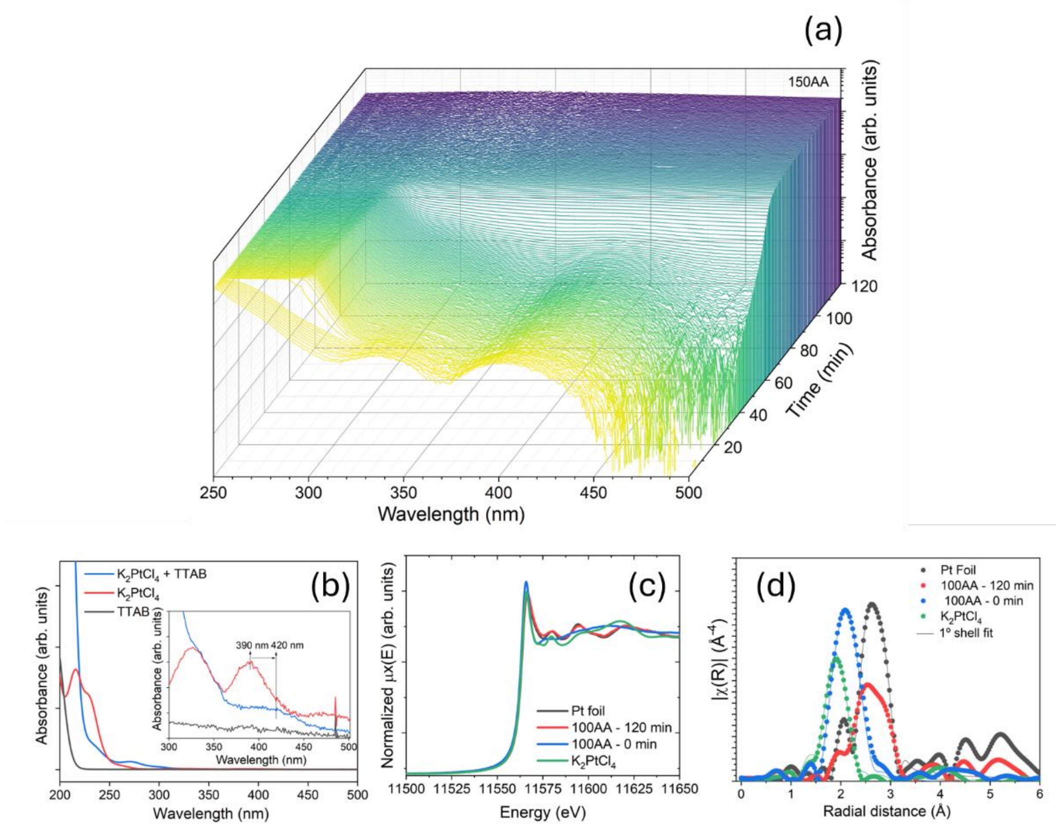

Figure. S3. (a) In situ UV-Vis data, showing the initial absorption band shift from approximately 390 to 420 nm. (b) Comparison of UV-Vis spectra for K_2PtCl_4 solution and K_2PtCl_4 + TTAB, highlighting the band position shift. (c) EXAFS spectra and (d) Fourier-transformed EXAFS signals for Pt foil, K_2PtCl_4 salt, and the initial and final 100AA samples.

Table S1. EXAFS data fitting results, showing the average distances and coordination numbers in the first coordination shell of platinum.

Sample	Atom pair	Coordination number, N	Interatomic distance, R (Å)	Debye-Waller factor, σ^2 (Å^2)	Threshold energy, E_0 (eV)	Amplitude reduction factor, S_0^2	R-factor	Reduced X^2
**Pt foil**	Pt-Pt	12	2.77 +/- 0.01	0.00452 +/- 0.000283	8.03 +/- 0.041	0.82 +/- 0.041	0.0048	1194.34
**100AA, 120 min**	Pt-Pt	10.04 +/- 0.91	2.76 +/- 0.01	0.00507 +/- 0.000671	7.84 +/- 0.68	0.82	0.01224	40.73
**K_2PtCl_4**	Pt-Cl	4	2.30 +/- 0.01	0.00181 +/- 0.000963	8.65 +/- 1.27	0.72 +/- 0.075	0.015965	28088.20
**100AA, 0 min (K_2PtCl_4 + TTAB)**	Pt-Br	4.55 +/- 0.81	2.43 +/- 0.01	0.0024558 +/- 0.001105	4.34 +/- 2.49	0.72	0.0178664	124.92

Figure S4a-d shows TEM images of Pt nanoparticles from samples 0AA, 50AA, 100AA, and 150AA, respectively. The nanoparticles synthesized in the presence of TTAB exhibit a hierarchical porous structure.

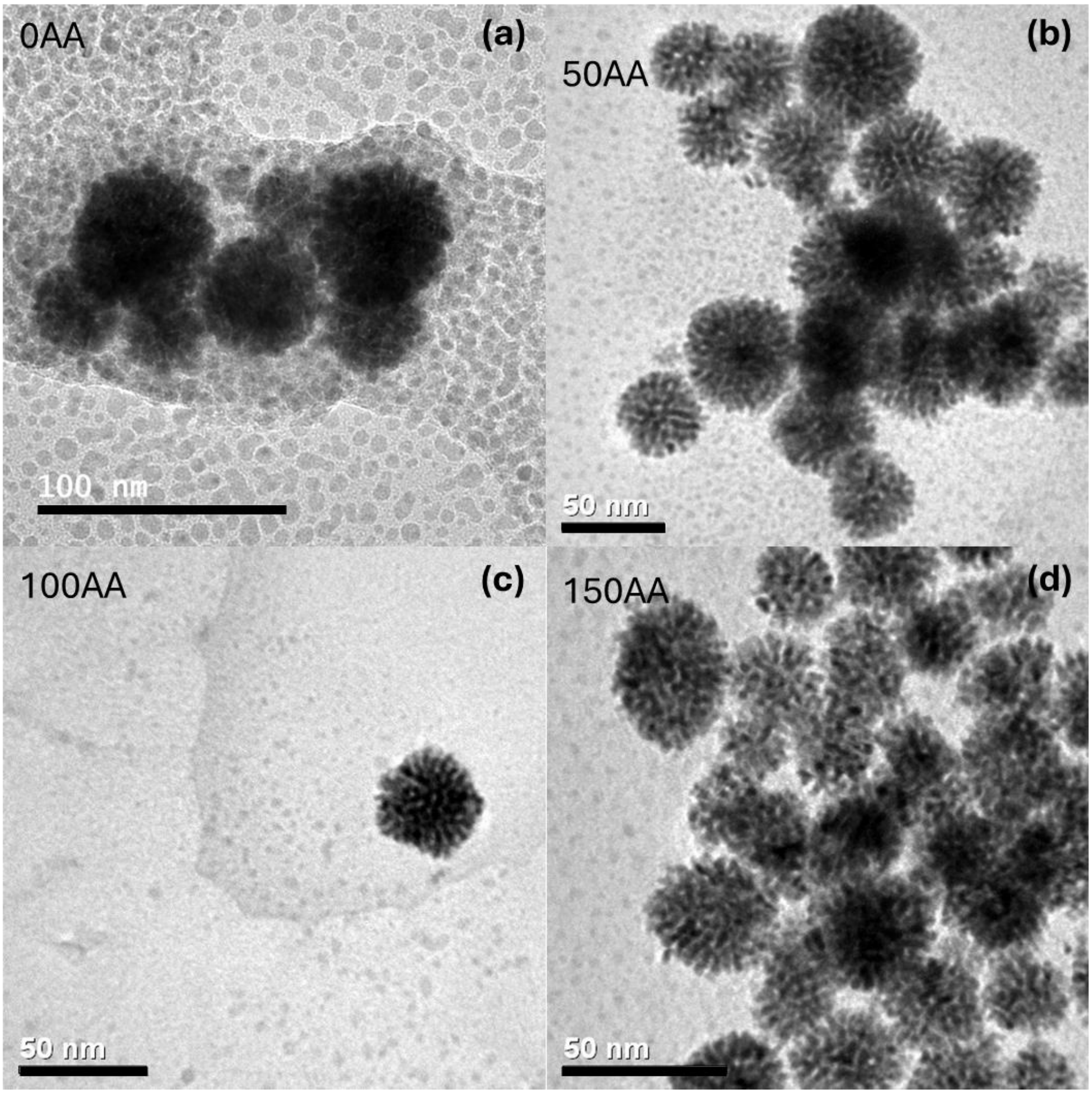

Figure. S4. TEM images of Pt nanoparticles from samples (a) 0AA, (b) 50AA, (c) 100AA, and (d) 150AA. The NP synthesized in presence of TTAB exhibit similar characteristics (porous structures).

TEM images of the primary nanoparticles are shown in Figure S5a-c for samples 50AA, 100AA, and 150AA, respectively. Figure S5d-f shows particle size distribution histograms for samples 50AA, 100AA, and 150AA, respectively. The histograms clearly illustrate that the particle size decreased and the distribution became narrower, as the amount of surfactant increased.

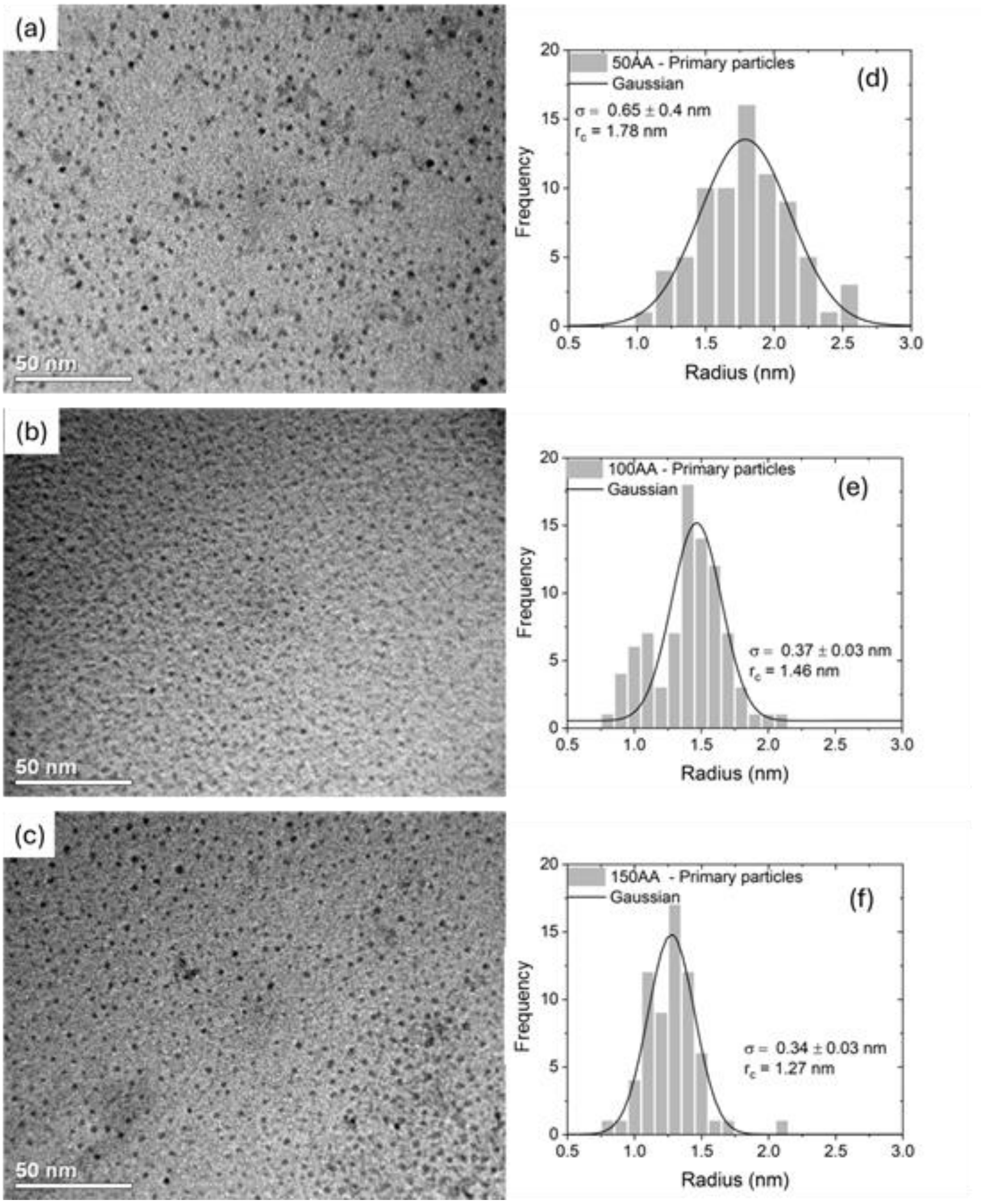

Figure. S5. TEM images of primary nanoparticles for samples 50AA (a), 100AA (b), and 150AA (c). Particle size distributions for samples 50AA (d), 100AA (e), and 150AA (f).

The scattering intensity around the correlation peak ($q < 1$ nm^{-1}), associated with the average inter-micellar distance, and the inter-micellar correlation distance is presented in Figure S6. Intensity increases linearly with increasing TTAB concentration while the correlation distance decreases.

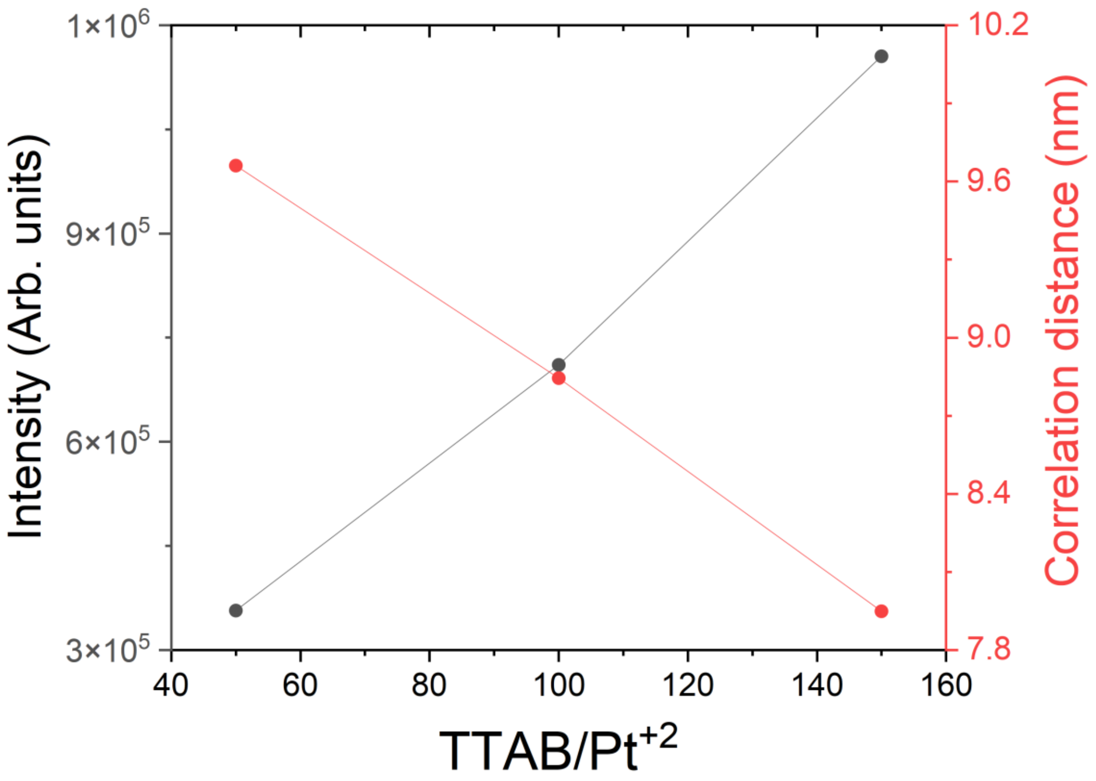

Figure. S6. Scattering intensity profiles in the low-q region ($q < 1$ nm^{-1}) showing the progressive increase in intensity around the inter-micellar correlation peak with increasing TTAB concentration. This effect is attributed to the decrease in the Debye screening length due to higher ionic strength (bromide ions), leading to weakened electrostatic repulsion and reduced inter-micellar distances.

REFERENCES

(1) Gómez, J. F.; Briosio, M. A.; Machado, J. D.; Sánchez, J. L.; Borges, R. New Approaches for Analysis of Amperometrical Recordings. *Ann. N. Y. Acad. Sci.* **2002**, *971* (1), 647–654. https://doi.org/10.1111/j.1749-6632.2002.tb04544.x.

(2) Ilavsky, J.; Jemian, P. R. Irena : Tool Suite for Modeling and Analysis of Small-Angle Scattering. *J. Appl. Crystallogr.* **2009**, *42* (2), 347–353. https://doi.org/10.1107/S0021889809002222.

(3) OriginLab Corporation. *OriginPro, Version 2024*; Northampton, MA, USA.
